# Heterogeneous acidic catalysts for the tetrahydropyranylation of alcohols and phenols in green ethereal solvents

**DOI:** 10.3762/bjoc.14.141

**Published:** 2018-07-03

**Authors:** Ugo Azzena, Massimo Carraro, Gloria Modugno, Luisa Pisano, Luigi Urtis

**Affiliations:** 1 Dipartimento di Chimica e Farmacia, Università degli Studi di Sassari, via Vienna 2, 07100 – Sassari, Italy

**Keywords:** green chemistry, green solvents, heterogeneous catalysts, multistep reactions, tetrahydropyranylation

## Abstract

The application of heterogeneous catalysis and green solvents to the set up of widely employed reactions is a challenge in contemporary organic chemistry. We applied such an approach to the synthesis and further conversion of tetrahydropyranyl ethers, an important class of compounds widely employed in multistep syntheses. Several alcohols and phenols were almost quantitatively converted into the corresponding tetrahydropyranyl ethers in cyclopentyl methyl ether or 2-methyltetrahydrofuran employing NH_4_HSO_4_ supported on SiO_2_ as a recyclable acidic catalyst. Easy work up of the reaction mixtures and the versatility of the solvents allowed further conversion of the reaction products under one-pot reaction conditions.

## Introduction

Due to their general stability to a wide range of reagents and ease of removal, tetrahydropyranyl (THP) ethers are widely employed in multistep organic synthesis for the protection of hydroxy derivatives [1–2]. In addition, they can be converted into a wealth of useful functional groups [2–3], and found employment as fragrances or pro-fragrances in everyday life [4–7]. Although a lot of work has been devoted to the search of low impact, heterogeneous and recyclable catalysts to promote THP ethers synthesis [2,8–14], to the best of our knowledge no attention was dedicated to their employment in low impact solvents.

From this point of view, it is worth noting that although ethers are the solvents of choice for reactions involving highly polar nucleophilic reagents such as organolithium and organomagnesium compounds or aluminium hydrides, tetrahydropyranylation reactions are usually run in hydrocarbons, chloroalkanes or dipolar aprotic solvents [1–2,8–15], thus affording a paradigmatic example of the ironic Murphy’s Law of Solvents, recently stated by Jessop et al. [16]: “The best solvent for any process step is bad for the next step”.

Starting from these premises and following our interest in the development of reaction procedures in low impact solvents [17–18], we report here on the tetrahydropyranylation of alcohols and phenols in cyclopentyl methyl ether (CPME) and 2-methyltetrahydrofuran (2-MeTHF) in the presence of heterogeneous acidic catalysts. Indeed, both CPME [19–22] and 2-MeTHF [21–25] are characterized by relatively high boiling points, a narrow explosion range, hydrophobicity, easy drying and recovery possibilities. Additionally, CPME is produced via a 100% atom economical reaction [19–20], whilst 2-MeTHF is accessible from renewable resources [21,23]. Finally, it is worth noting that besides being moderately irritant, both solvents are characterized by low toxicities and are considered negative for genotoxicity and mutagenicity [26–28]. Due to these environmentally friendly characteristics, CPME and 2-MeTHF appear as versatile green alternatives to ethereal solvents such as tetrahydrofuran, dioxane, diethyl ether or methyl *tert*-butyl ether.

Aiming to the development of a particularly practical and environmentally friendly procedure to the generation of THP ethers, we devoted our efforts to the employment of heterogeneous acidic catalysts [29–30] in order to set up conditions allowing easy processing of the reaction mixtures and, possibly, the recovery and recycling of the catalyst.

## Results and Discussion

**Scheme 1:**
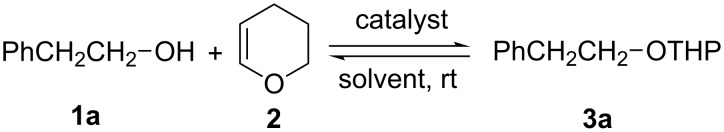
Synthesis of THP ether **3a**.

Taking 2-phenylethanol (**1a**) as a model compound, we investigated its conversion into the corresponding THP ether **3a** by reacting 4 M solutions of this alcohol with a slight excess (1.1 equiv) of 3,4-dihydro-2*H*-pyran (**2**, DHP) in the presence of several acidic heterogeneous catalysts (Scheme 1 and Table 1). The recovered reaction mixtures were very simply elaborated by filtering the catalyst followed by evaporation of the solvent in vacuo.

**Table 1:** Synthesis of 2-(2-phenylethoxy)tetrahydro-2*H*-pyran (**3a**).

entry	solvent	catalyst (%)	**3a**/**1a** (%)^b^

1	CPME or 2-MeTHF	none	<5:>95
2	CPME or 2-MeTHF	NH_4_Cl	<5:>95
3	CPME or 2-MeTHF	NH_4_Br	<5:>95
4	CPME or 2-MeTHF	NH_4_H_2_PO_4_	<5:>95
5	CPME or 2-MeTHF	NH_4_HSO_4_	94:6
6	CPME	NaHSO_4_	93:7
7	CPME	KHSO_4_	93:7
8	2-MeTHF	Amberlyst 15	>95:<5
9	CPME or 2-MeTHF	Montmorillonite K10^c^	>95:<5
10	CPME	NH_4_HSO_4_@SiO_2_^d,e^	>95:<5
11	2-MeTHF	NH_4_HSO_4_@SiO_2_	>95:<5
12	CPME	NH_4_HSO_4_@SiO_2_^f^	93:7

^a^All reactions were run at rt during 4 h in the presence of the catalyst (3 mol % of **1a**, unless otherwise indicated). ^b^As determined by ^1^H NMR analyses of crude reaction mixtures; no other product was detected besides **1a**. ^c^**1a**/catalyst = 3 wt %. ^d^Comparable results were obtained after 4 times recycling of the catalyst. ^e^No reaction was observed in the presence of a comparable amount of carefully dried SiO_2_. ^f^3 mol ‰ of **1a**.

No reaction occurred in both solvents in the absence of an acidic catalyst as well as in the presence of several ammonium salts, i.e., NH_4_Cl, NH_4_Br and NH_4_H_2_PO_4_ (Table 1, entries 1–4). On the other hand, inorganic salts with a relatively higher acidity, i.e., NH_4_HSO_4_, NaHSO_4_ and KHSO_4_ [31], as well as Amberlyst 15 and Montmorillonite K10, efficiently promoted practically quantitative conversion of the starting material into the corresponding tetrahydropyranyl ether **3a** within a few hours in both solvents (Table 1, entries 5–9).

Good results were also obtained with a 25 wt % dispersion of NH_4_HSO_4_ over SiO_2_ (NH_4_HSO_4_@SiO_2_, Table 1, entries 10 and 11). Additionally, the supported catalyst, which can be stored in a desiccator for several months with no detrimental effects, is particularly easy to recover and was recycled up to 4 times with no evident loss of activity (Table 1, entry 10). Its robustness was further assessed by the good qualitative matching between the IR spectra of the fresh and recycled catalysts (see Supporting Information File 1, Figure S1). Additionally, it is worth noting that a comparable result was obtained by reducing the amount of catalyst to one tenth (3 mol ‰ of **1a**, Table 1, entry 12).

Finally, both CPME and 2-MeTHF proved stable towards such an acidic supported catalyst, as established by the absence of any decomposition product within the reaction mixtures, as determined by ^1^H, ^13^C NMR and gas-liquid chromatography analyses [32].

**Scheme 2:**
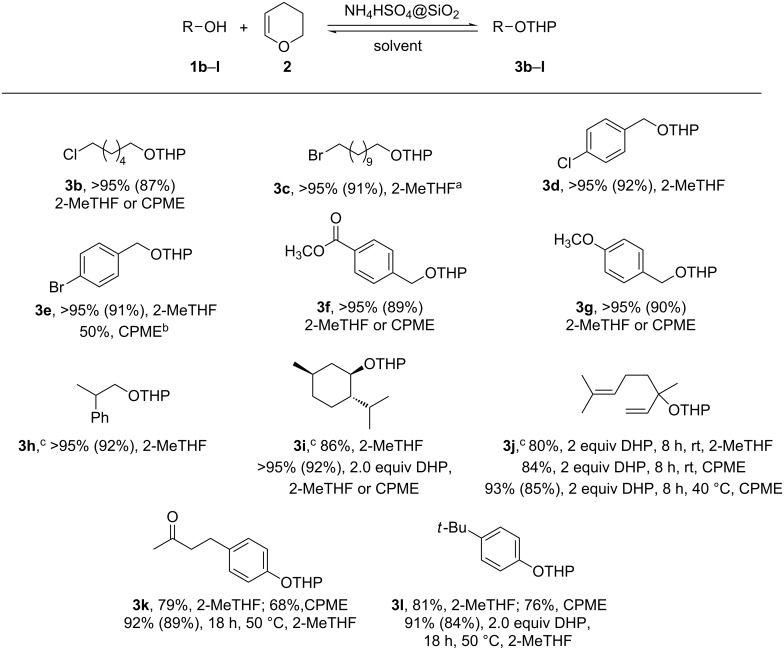
Synthesis of THP ethers **3b**–**l** in the presence of NH_4_HSO_4_@SiO_2_. All reactions were run at rt, in the presence of 1.1 equiv of **2** and 3 mol ‰ of catalyst during 4 h, unless otherwise indicated; percentages represent conversion of the starting materials as determined by ^1^H NMR; isolated yields are reported in brackets; no other product, besides starting materials, was detected. ^a^No reaction in the presence of comparable amounts of NH_4_Br or SiO_2_. ^b^**1e** is almost insoluble in CPME. ^c^1:1 mixture of diastereoisomers.

Due to the ease of the preparation from particularly cheap starting materials, the ease of handling and its high activity, we further investigated the ability of NH_4_HSO_4_@SiO_2_ to catalyze the synthesis of different THP ethers in low impact ethereal solvents. Accordingly, the reactions were performed in the presence of 3 mol ‰ of the catalyst and were successfully applied to the synthesis of a series of THP ethers of functionalized and non-functionalized alcohols and phenols, including some known fragrances (**3g**–**j** and **3l**) [4–7], as illustrated in Scheme 2.

Conversions exceeding 95% were achieved under the mild conditions illustrated in Scheme 2 with primary aliphatic and benzylic alcohols **1b**–**h**. A notable exception is the low conversion of 4-bromobenzyl alcohol **1e** in CMPE, most probably due to the low solubility of the starting material in this solvent. This disadvantage was easily overcome by performing the reaction in 2-MeTHF.

More demanding conditions (2.0 equiv of **2**) were required to obtain good conversions of a secondary alcohol, i.e, (1*R*,2*S*,5*R*)-2-isopropyl-5-methylcyclohexanol ((−)-menthol, **1i**). The reaction afforded an almost 1:1 mixture of only two diastereoisomers as determined by ^1^H and ^13^C NMR analysis of the crude reaction mixture, thus suggesting that it occurs with no epimerization at C1.

**Scheme 3:**
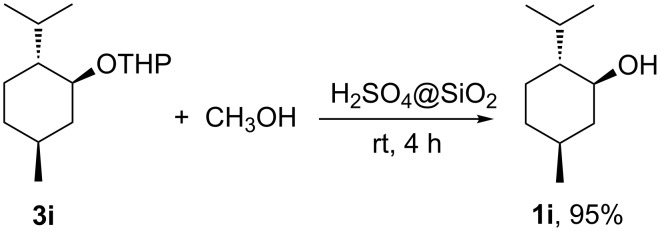
Deprotection of THP ether **3i**.

This result was confirmed by submitting compound **3i** to acid-catalyzed deprotection with H_2_SO_4_@SiO_2_ (25 wt % [33] as depicted in Scheme 3). Alcohol **1i**, was recovered as a single stereoisomer, identical to the commercially available starting material, as determined by ^1^H and ^13^C NMR analysis of the crude reaction mixture (see Supporting Information File 1).

Finally, the tetrahydropyranylation of a tertiary alcohol **1j** as well as of phenols **1k** and **1l** required, besides the employment of 2.0 equiv of DHP, relatively longer reaction times and higher temperatures. It is worth noting that under our mild reaction conditions we did not observe any isomerisation of the acid-sensitive allylic alcohol **1j** [34].

To stress the usefulness of a tetrahydropyranylation reaction performed in a green ethereal solvent and in the presence of a heterogeneous acidic catalyst, we realized two one-pot procedures employing either 2-MeTHF or CPME as a solvent.

**Scheme 4:**
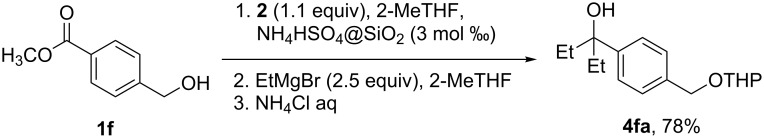
One-pot synthesis of 3-[4-(tetrahydro-2*H*-pyran-2-yl)oxymethylphenyl]-3-pentanol (**4fa**).

Accordingly, the mixture obtained by reacting **1f** with 1.1 equiv of **2** and 3 mol ‰ of NH_4_HSO_4_@SiO_2_ in 2-MeTHF under dry Ar was filtered and dropwise added to a vigorously stirred freshly prepared solution of EtMgBr in the same solvent at rt. Aqueous work-up and flash chromatography afforded the desired tertiary alcohol **4fa** in 78% yield (Scheme 4).

**Scheme 5:**
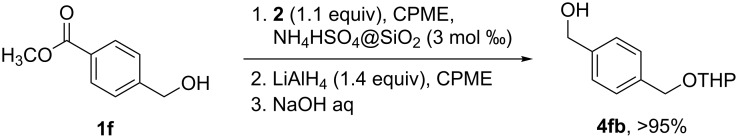
One-pot synthesis of 4-(tetrahydro-2*H*-pyran-2-yloxymethyl)benzyl alcohol (**4fb**).

Under similar conditions, protection of **1f** in CPME, followed by filtration and dropwise addition of the resulting solution to a suspension of LiAlH_4_ in the same solvent, afforded the monoprotected diol **4fb** in almost quantitative yield (Scheme 5).

## Conclusion

The above reported results show that several heterogeneous acidic catalysts efficiently promote the tetrahydropyranylation of an alcoholic model compound in low impact ethereal solvents under mild conditions. Further investigations show that NH_4_HSO_4_@SiO_2_, easily prepared from inexpensive starting materials, successfully catalyzes the protection of a variety of functionalized and non-functionalized alcohols and phenols, including an optically pure alcohol with no detrimental effects on its stereochemistry. Due to a particularly simple work-up procedure, NH_4_HSO_4_@SiO_2_ can be easily recovered and recycled several times. The easy removal of the acidic catalyst from the reaction mixtures and the versatility of the employed solvents allowed the successful further conversion of the reaction products with strong nucleophiles under one-pot conditions.

## Supporting Information

File 1 Full experimental details, copies of IR spectra of fresh and recycled NH_4_HSO_4_@SiO_2_ and copies of ^1^H and ^13^C NMR spectra.
