# Supplementary material for: Heterogeneous acidic catalysts for the tetrahydropyranylation of alcohols and phenols in green ethereal solvents
Source: Beilstein J Org Chem. 2018 Jul 3;14:1655–9. doi: 10.3762/bjoc.14.141 (PMC6036977; doi:10.3762/bjoc.14.141)
Supplement: File 1 — Full experimental details, copies of IR spectra of fresh and recycled NH4HSO4@SiO2 and copies of 1H and 13C NMR spectra. [file Beilstein_J_Org_Chem-14-1655-s001.pdf]

Supporting Information

for

Heterogeneous acidic catalysts for the tetrahydropyranylation of alcohols and phenols in green ethereal solvents

Ugo Azzena*, Massimo Carraro, Gloria Modugno, Luisa Pisano and Luigi Urtis

Address: Dipartimento di Chimica e Farmacia, Università degli Studi di Sassari, via Vienna 2, 07100 – Sassari, Italy

Email: Ugo Azzena - ugo@uniss.it

*Corresponding author

Full experimental details, copies of IR spectra of fresh and recycled $NH_4HSO_4@SiO_2$ and copies of ^{1}H and ^{13}C NMR spectra

General Information. All reagents and solvents were purchased from commercial suppliers and used without further purification, unless otherwise indicated. $H_2SO_4@SiO_2$ (25 wt %) was prepared as described in ref. [1]. ^{1}H NMR (400 MHz) and ^{13}C NMR (100 MHz) spectra were recorded with a Bruker Ascend 400 spectrometer in $CDCl_3$ solutions with $SiMe_4$ as internal standard. The FTIR spectra were recorded from KBr pellets on a Fourier Transform Infrared Spectrophotometer

on a Jasco FT/IR-480 Plus. Gas chromatographic analyses were recorded on a Agilent 6890N Network GC System. The HRMS spectra were acquired on a Thermo Finnigan Q Exactive instrument with API-HESI source. Samples were introduced as 0.1 mg/L solutions in MS grade methanol with a 5 µL/min flow and the following source parameters: positive polarity; Sheath gas flow rate: 5 a.u.; Aux gas flow rate: 3 a.u.; Sweep gas flow rate: 0 a.u.; Spray voltage: 3.50 kV; Capillary temperature: 250 °C; S-lens RF level: 60,0 V; Aux gas heater temperature: 0 °C.

Preparation of the NH_4HSO_4@SiO_2 catalyst. *Preparation of NH_4HSO_4@SiO_2*

NH_4HSO_4@SiO_2 (25wt %) was prepared by wet impregnation. 17.4 g of SiO_2 (column chromatographic grade, 60 Å, 200–400 mesh) were added to a stirred solution of 5.8 g (50 mmol) of NH_4HSO_4 dissolved in 30 mL of H_2O, and the suspension was stirred at 50 °C during 1 h, followed by solvent evaporation under reduced pressure. The resulting white powder was dried at 120 °C for 48 h, then transferred and stored in a desiccator over $CaCl_2$. IR (cm^{-1}): 3448, 3172, 1641, 1507, 1400, 1190, 1098, 971, 879, 600.

General procedure: Tetrahydropyranylation reactions were carried out in a round bottom flask equipped with a reflux condenser, a $CaCl_2$ tube and a magnetic stirring bar. To a solution of 1.0×10^{-2} mol of compound **1** in 2.5 mL of the solvent (CPME or 2-MeTHF) were added 1.1×10^{-2} mol (0.93 g, 1.0 mL) of 2,3-dihydropyran (**2**) and the required amount of the catalyst (3.3×10^{-4} or 3.3×10^{-5} mol; see Table 1 and Schemes 1 and 2). The resulting mixtures were vigorously stirred for the reported time at the reported temperature (Table 1 and Schemes 1 and 2). The crude mixtures were filtered, the solvent evaporated and the crude product analyzed by ^{1}H-NMR spectroscopy to determine the conversion of compound **1**, then purified and characterized as reported below.

2-(Phenylethoxy)tetrahydro-2*H*-pyran (3a) [2]**.** Pale yellow oil, purified by flash chromatography, (AcOEt/Petroleum Ether/Et_3N = 3/7/0.1, R_f = 0.56); ^{1}H-NMR (400 MHz, $CDCl_3$) δ 1.46-1.95 (m, 6H), 2.95 (t, *J* = 7.2 Hz, 2H), 3.42-3.48 (m, 1H), 3.63 (q, *J* = 8.0 Hz, 1H), 3.74-3.81 (m, 1H), 3.96 (q, *J* = 8.0 Hz, 1H), 4.63 (bs, 1H), 7.18-7.32 (m, 5H); ^{13}C NMR (100 MHz, $CDCl_3$) δ 19.4, 25.4, 30.7, 36.4, 62.1, 68.3, 98.7, 126.1, 128.2, 129.0, 139.1.

2-(6-Chlorohexyloxy)tetrahydro-2*H*-pyran (3b) [3,4]**.** Pale yellow oil, purified by fractional distillation, bp 115 °C/ 1Torr, Lit. bp 107 °C/0.25 Torr [4]; ^{1}H-NMR (400 MHz, $CDCl_3$) δ 1.35-1.90 (14H, m), 3.36-3.42 (1H, m), 3.45-3.56 (3 H, m), 3.70-3.78 (1H, m), 3.84-3.90 (1H, m), 4.57 (s, 1H); ^{13}C NMR (100 MHz, $CDCl_3$) δ 19.7, 25.5, 25.6, 26.7, 29.6, 30.8, 32.6, 45.0, 62.4, 67.4, 98.9.

2-(11-Bromoundecyloxy)tetrahydro-2*H*-pyran (3c) [5]**.** Colourless oil, purified by flash chromatography, (AcOEt/Petroleum Ether/Et_3N = 0.5/9.5/0.1, R_f= 0.52); ^{1}H-NMR (400 MHz, $CDCl_3$) δ 1.28-1.46 (m, 14H), 1.47-1.64 (m, 6H), 1.65-1.76 (m, 1H), 1.78-1.90 (m, 3H), 3.34-3.43 (m. 3H), 3.48-3.52 (m, 1H), 3.73 (q, 1H, *J* = 7.6 Hz), 3.83-3.90 (m, 1H), 4.58 (s, 1H); ^{13}C NMR (100 MHz, $CDCl_3$) δ 19.7,19.7, 25.5, 26.2, 28.1, 28.7, 29.4, 29.4, 29.5, 29.7, 30.8, 32.8, 34.0, 62.3, 67.7, 98.8.

2-(4-Chlorobenzyloxy)tetrahydro-2*H*-pyran (3d) [2]. Colourless oil, purified by flash chromatography, (AcOEt/Petroleum Ether/Et_3N = 3/7/0.1, R_f = 0.58); ^{1}H-NMR (400 MHz, $CDCl_3$) δ 1.50-1.67 (4H, m), 1.68-1.77 (1H, m), 1.80-1.93 (1H, m), 3.50-3.58 (1H, m), 3.90 (1H, t, *J* = 9.6 Hz), 4.48 (1H, d, *J* = 12.0 Hz), 4.69 (1H, bs), 4.75 (1H, d, *J* = 12.0 Hz), 7.31 (4H, s); ^{13}C NMR (100 MHz, $CDCl_3$) δ 19.3, 25.4, 30.5, 62.1, 67.9, 97.7, 128.4, 129.0, 133.1, 136.8.

2-(4-Bromobenzyloxy)tetrahydro-2*H*-pyran (3e) [6]. Colourless oil, purified by flash chromatography, (AcOEt/Petroleum Ether/Et_3N = 3/7/0.1, R_f = 0.61); ^{1}H-NMR (400

MHz, $CDCl_3$) δ 1.51-1.72 (4H, m), 1.72-1.82 (1H, m), 1.83-1.95 (1H, m), 3.53-3.62 (1H, m), 3.88-3.97 (1H, m), 4.49 (1H, d, *J* = 12.4 Hz), 4.69 (1H, t, *J* = 3.2 Hz), 4.75 (1H, d, *J* = 12.4 Hz), 7.26-7.29 (2H, m), 7.50-7.52 (2H, m); ^{13}C NMR (100 MHz, $CDCl_3$) δ 19.3, 25.4, 30.5, 62.1, 68.0, 97.7, 121.3, 129.4, 131.4, 137.3.

Methyl 4-[(tetrahydro-2*H*-pyran-2-yl)oxymethyl]benzoate (3f) [7]. Colourless oil, purified by flash chromatography, (AcOEt/Petroleum Ether/Et_3N = 2/8/0.1, R_f = 0.48); ^{1}H-NMR (400 MHz, $CDCl_3$) δ 1.42-2.00 (6H, m), 3.52-3.58 (1H, m), 3.87-3.99 (1H, m), 3.91 (3H, s), 4.56 (1H, d, *J* = 13.2 Hz), 4.72 (1H, bs), 4.84 (1H, d, *J* = 13.2 Hz) 1 H 7.42-7.44 (2 H, m), 8.01-8.03 (2H, m); ^{13}C NMR (100 MHz, $CDCl_3$) δ (ppm) 19.4, 25.5, 30.6, 52.2, 62.3, 68.3, 76.8, 77.2, 77.5, 98.1, 127.3, 129.3, 129.8, 143.8, 167.1.

2-(4-Methoxybenzyloxy)tetrahydro-2*H*-pyran (3g) [8]. Colourless oil, purified by flash chromatography, (AcOEt/Petroleum Ether/Et_3N = 2/8/0.1, R_f = 0.53); ^{1}H-NMR (400 MHz, $CDCl_3$) δ 1.48-1.66 (4H, m), 1.67-1.77 (1H, m), 1.80-1.89 (1H, m), 3.50-3.58 (1H, m), 3.81 (3H, s), 3.88-3.96 (1H, m), 4.44 (1H, d, *J* = 11.6 Hz), 4.69 (1H, t, *J* = 3.6 Hz), 4.73 (1H, d, *J* = 11.6 Hz), 6.86-6.90 (2H, m), 7.27-7.32 (2H, m); ^{13}C NMR (100 MHz, $CDCl_3$) δ 19.4, 25.5, 30.6, 55.2, 62.1, 68.4, 97.4, 113.7, 129.4, 130.3, 159.1.

2-(2-Phenylpropoxy)tetrahydro-2*H*-pyran (3h) [9]**.** Colourless oil, 1:1 mixture of diastereoisomers, purified by flash chromatography (AcOEt/Petroleum Ether/Et_3N = 3/7/0.1, R_f = 0.60); ^{1}H-NMR (400 MHz, $CDCl_3$) δ 1.31 (1.5H, d *J* = 5.2 Hz), 1.33 (1.5 H, d, *J* = 5.2 Hz), 1.42-1.76 (6H, m), 3.04 (1H, sex, *J* = 6.8 Hz), 3.37-3.54 (2H, m), 3.63 (0.5H, m), 3.73-3.89 (1.5H, m), 4.53 (0.5H, t, *J* = 3.6 Hz), 4.59 (0.5H, t, *J* = 3.6 Hz), 7.16-7.32 (5H, m) (fractional integrals due to the presence of two diastereomers); ^{13}C NMR (100 MHz, $CDCl_3$) δ 18.3, 18.4, 19.3, 19.4, 25.5, 25.5, 30.6,

40.0, 40.0, 61.9, 62.0, 73.0, 73.2, 98.5, 98.8, 126.2, 126.2, 127.4, 127.4, 128.2, 128.2, 144.5, 144.6.

2-[(1*R*,2*S*,5*R*)-5-Methyl-2-(1-methylethyl)cyclohexyloxy]tetrahydro-2*H*-pyran (3i) [10,11]**.** Colourless oil, 1:1 mixture of diastereoisomers, purified by fractional distillation, bp 132 °C/ 1 Torr, Lit. bp 100 °C/ 5 Torr [11]; ^{1}H-NMR (400 MHz, $CDCl_3$) δ 0.77 (1.5 H, d, J = 6.8 Hz), 0.80 (1,5H, d, J = 7.6 Hz), 0.81-1.11 (8,5H, m), 1.18-1.30 (0.5H, m), 1.30-1.90 (9.5H, m), 2.03-2.19 (1.5H, m), 2.35 (0.5H, pentd, J = 10.8, 2.4 Hz), 3.31 (0.5H, td, J = 10.8, 4.4 Hz), 3.43-3.53 (1.5H, m), 3.84-3.92 (0.5H, m), 3.93-4.00 (0.5H, m), 4.57-4.61 (0.5H, m), 4.77-4.81 (0.5H, m) (fractional integrals due to the presence of two diastereomers); ^{13}C NMR (100 MHz, $CDCl_3$) δ 15.6, 16.3, 19.7, 20.3, 21.2, 21.2, 22.2, 22.4, 23.0, 23.3, 25.2, 25.5, 25.6, 25.6, 31.2, 31.3, 31.4, 31.8, 34.4, 34.6, 40.1, 43.6, 48.1, 48.9, 62.4, 63.0, 74.1, 79.9, 94.3, 101.3.

2-[(1-Ethenyl-1,5-dimethyl-4-hexen-1-yl)oxy]tetrahydro-2*H*-pyran (3j) [12,13]. Colourless oil, 1:1 mixture of diastereoisomers, purified by fractional distillation, bp 110-115 °C/ 1Torr, Lit. bp 80-83 °C/0.04 Torr [13]; ^{1}H-NMR (400 MHz, $CDCl_3$) δ 1.27 (1.5H, s), 1.32 (1.5H, s), 1.46-1.71 (6H, m) 1.78-2.11 (3H, m), 3.39-3.48 (1H, m), 3.91-3.99 (1H, m), 4.62-4.68 (1.5H, m), 5.07-5.19 (2.5H, m), 5.80 (0.5H, dd, J = 17.2, 11.6 Hz), 5.95 (0.5H, dd, J = 17.6, 10.8 Hz) (fractional integrals due to the presence of two diastereomers); ^{13}C NMR (100 MHz, $CDCl_3$) δ 17.5, 20.5, 20.6, 22.5, 22.6, 22.8, 23.5, 25.4, 25.5, 25.6, 32.2, 32.4, 40.2, 41.7, 63.0, 63.2, 78.4, 78.6, 94.1, 94.5, 113.4, 114.3, 124.6, 131.2, 143.2, 143.6.

4-[4-[(Tetrahydro-2*H*-pyran-2-yl)oxy]phenyl]butan-2-one (3k) [14]**.** Colourless oil, purified by aqueous basic work up; ^{1}H-NMR (400 MHz, $CDCl_3$) δ 1.57-1.66 (3H, m), 1.84-1.88 (2H, m), 1.97-2.02 (1H, m), 2.71 (2H, t, J = 7.2 Hz), 2.83 (2H, t, J = 7.2 Hz), 3.54-3.64 (1H, m), 3.90 (1H, t, J = 10.4 Hz), 5.36 (1H, bs), 6.95-6.97 (2H, m), 7.07-

7.09 (2H, m); ^{13}C NMR (100 MHz, $CDCl_3$) δ 18.8, 25.2, 28.9, 30.0, 30.3, 45.3, 62.0, 96.4, 116.5, 129.1, 133.9, 155.4, 208.0.

2-(4-*tert*-Butylphenoxy)tetrahydro-2*H*-pyran (3l) [15,16]**.** Colourless oil, purified by aqueous basic work up; ^{1}H-NMR (400 MHz, $CDCl_3$) δ 1.30 (9H, s), 1.56-1.74 (3H, m) 1.82-1.88 (2H, m), 1.96-2.08 (1H, m), 3.56-3.64 (1H, m), 3.89-3.98 (1H, m), 5.39 (t, 1 H, *J* = 3.2 Hz), 6.96-7.00 (2H, m), 7.28-7.32 (2H, m); ^{13}C NMR (100 MHz, $CDCl_3$) δ 18.9, 25.3, 30.5, 31.5, 34.1, 62.0, 96.5, 115.9, 126.1, 144.2, 154.8.

One-pot two-step synthesis of 3-[4-(tetrahydro-2*H*-pyran-2-yl)oxymethylphenyl]-3-pentanol (4fa). THP ether **3f** was prepared according to the general procedure by reacting 4-carboxymethylbenzyl alcohol **1f** (0.84 g, 5.0 mmol) with DHP (0.5 mL, 0.46 g, 5.5 mmol) under dry Ar in a two-necked flask equipped with reflux condenser and magnetic stirrer in dry 2-MeTHF (1.2 mL, distilled from Na/K alloy under dry Ar) in the presence of 3 mol ‰ of NH_4HSO_4@SiO_2 (1.5×10^{-5} mol of NH_4HSO_4, 25 wt %, 7.0 mg). After 4 h stirring at rt, the reaction mixture was filtered under dry Ar and dropwise added to a 2.5 M solution of EtMgBr in dry 2-MeTHF (5.0 mL, 2.5 equiv). The resulting mixture was stirred at reflux during 2 h, chilled at 0 °C, then quenched by slow dropwise addition of sat. NH_4Cl (37.5 mL). The organic phase was separated, the aqueous phase extracted with 2-MeTHF (2 × 10 mL) and the reunited organic phases were washed with sat. NH_4Cl (3 × 10 mL), H_2O (10 mL), dry (Na_2SO_4), filtered and evaporated to afford a colourless oil which was purified by flash chromatography, (AcOEt/Petroleum Ether/Et_3N = 4/6/0.1, R_f = 0.61); HRMS (ESI) m/z: calc per $C_{17}H_{26}O_3K^+$ = 317.15135 $[M^+K]^+$, found 317.15161; ^{1}H-NMR (400 MHz, $CDCl_3$) δ 1.76 (6H, t, *J* = 7.4 Hz), 1.50-1.94 (11H, m), 3.52-3.60 (1H, m), 3.89-3.97 (1H, m), 4.49 (1H, d, *J* = 12.0 Hz), 4.72 (1H, t, *J* = 3.2 Hz), 4.79

(1H, d, J = 12.0 Hz), 7.31-7.37 (4H, m); ^{13}C NMR (100 MHz, $CDCl_3$) δ 7.7, 19.3, 25.4, 30.5, 34.9, 62.0, 68.6, 77.2, 97.7, 125.5, 127.4, 136.1, 145.1.

One-pot two-steps synthesis of 4-(tetrahydro-2H-pyran-2-yloxymethyl)benzyl alcohol (4fb) [17]**.** THP ether **3f** was prepared as described in the preceding paragraph in dry CMPE (1.2 mL, distilled from Na/K alloy under dry Ar). Once filtered under dry Ar, it was diluted to 8.0 mL with dry CPME and added dropwise to a vigorously stirred suspension of $LiAlH_4$ (0.59 g, 15,5 mmol) in dry CPME (4 mL) at 0 °C. The reaction mixture was stirred during 24 hours at rt, then chilled to 0 °C and slowly quenched by dropwise addition of 20 mL of water (caution!). The resulting mixture was acidified with concentrated HCl (2.0 mL) and extracted with CPME (4 × 20 mL). The organic phases were collected, dried over K_2CO_3 and the solvent removed in vacuo to afford 2.31 g (10.4 mmol, yield 95%) of **4fb** as an oil that solidified upon standing at -18 °C. The compound was sufficiently pure for spectroscopic analysis.

^{1}H-NMR (400 MHz, $CDCl_3$) δ 1.48-1.79 (6H, m) 1.81-1.93 (1H, m), 3.52-3.59 (1H, m), 3.88-3.97 (1H, m), 4.51 (1H, d, J = 12.0 Hz), 4.68-4.72 (3H, m), 4.78 (1H, d, J = 12.0 Hz), 7.33-7.39 (4H, m); ^{13}C NMR (100 MHz, $CDCl_3$) 19.1, 25.2, 30.3, 61.9, 64.5, 64.4, 97.4, 126.8, 127.8, 137.2, 140.3.

Deprotection of 2-[(1*R*,2*S*,5*R*)-5-methyl-2-(1-methylethyl)cyclohexyloxy]tetrahydro-2*H*-pyran (3i). To a stirred solution of **3i** (1.00 g, 4.2 mmol) in CH_3OH (10 mL) was added H_2SO_4@SiO_2 (25 wt %, 49 mg, 1.3 × 10^{-4} mol, 3 mol %), and the resulting mixture was stirred at room temperature. After 4 h, H_2O (15 mL) was added and CH_3OH was removed on a rotary evaporator. The resulting mixture was extracted with AcOEt (3 × 5 mL), and the organic layer was washed with saturated aqueous $NaHCO_3$ (10 mL), H_2O (10 mL), dried (Na_2SO_4), and concentrated on a rotary evaporator to yield crude (−)-menthol (**1j**, 0.620 g, 4.0 ×

10^{-3}, 95%) which, without purification, was characterized as following [18,19]: ^{1}H-NMR (400 MHz, $CDCl_3$) δ 0.81 (3h, d, *J* = 7.2 Hz), 0-84-1.03 (8H, m), 1.07-1.15 (1H, m), 1.26-1.32 (1H, d, *J* = 8.8 Hz), 1.35-1.49 (1H, m), 1.58-1.70 (2H, m), 1.93-2.00 (1H, m), 2.17 (1H, eptd, *J* = 6.8, 2.8 Hz), 3.41 (1H, td, *J* = 10.4, 4.0 Hz); ^{13}C NMR (100 MHz, $CDCl_3$) 16.0, 21.0, 22.2, 23.1, 25.8, 31.6, 34.5, 45.0, 50.1, 71.4.

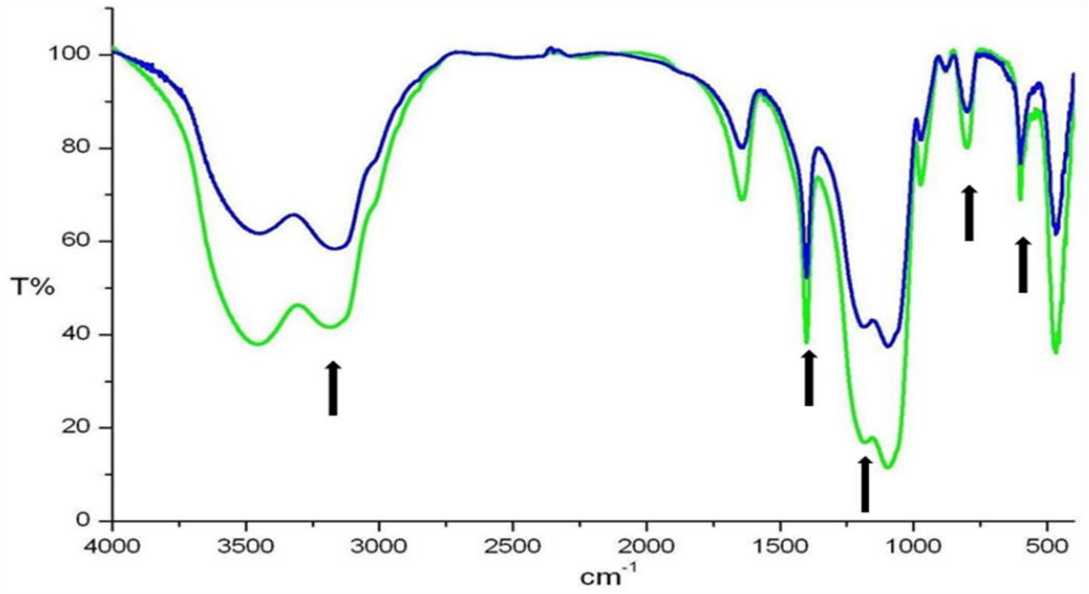

Figure S1. IR spectra of fresh (▬▬) and recycled (▬▬) NH_4HSO_4@SiO_2. Arrows indicate characteristic absorptions of NH_4HSO_4 at 3172, 1400, 1190, 879, and 600 cm^{-1}.

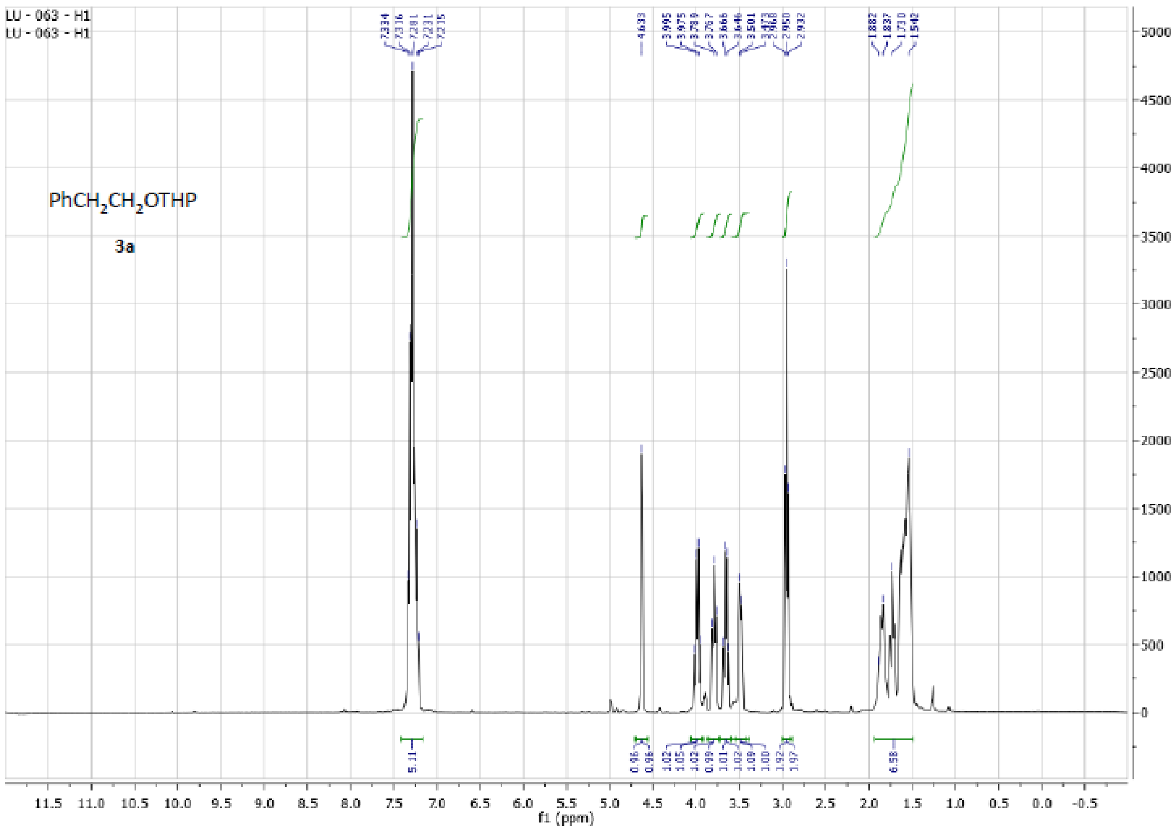

LU - 063 - H1
LU - 063 - H1
PhCH$_2$CH$_2$OTHP
3a
f1 (ppm)

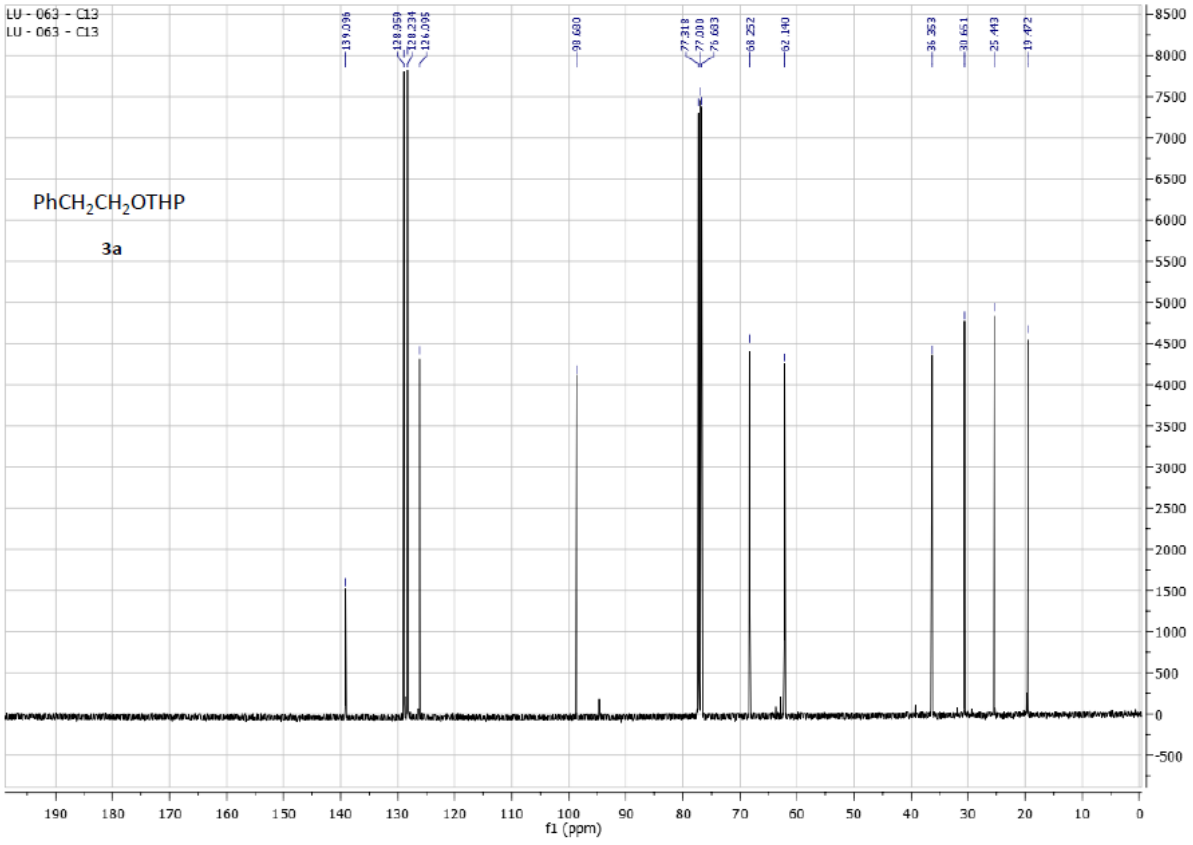

LU - 063 - C13
LU - 063 - C13
PhCH$_2$CH$_2$OTHP
3a
f1 (ppm)

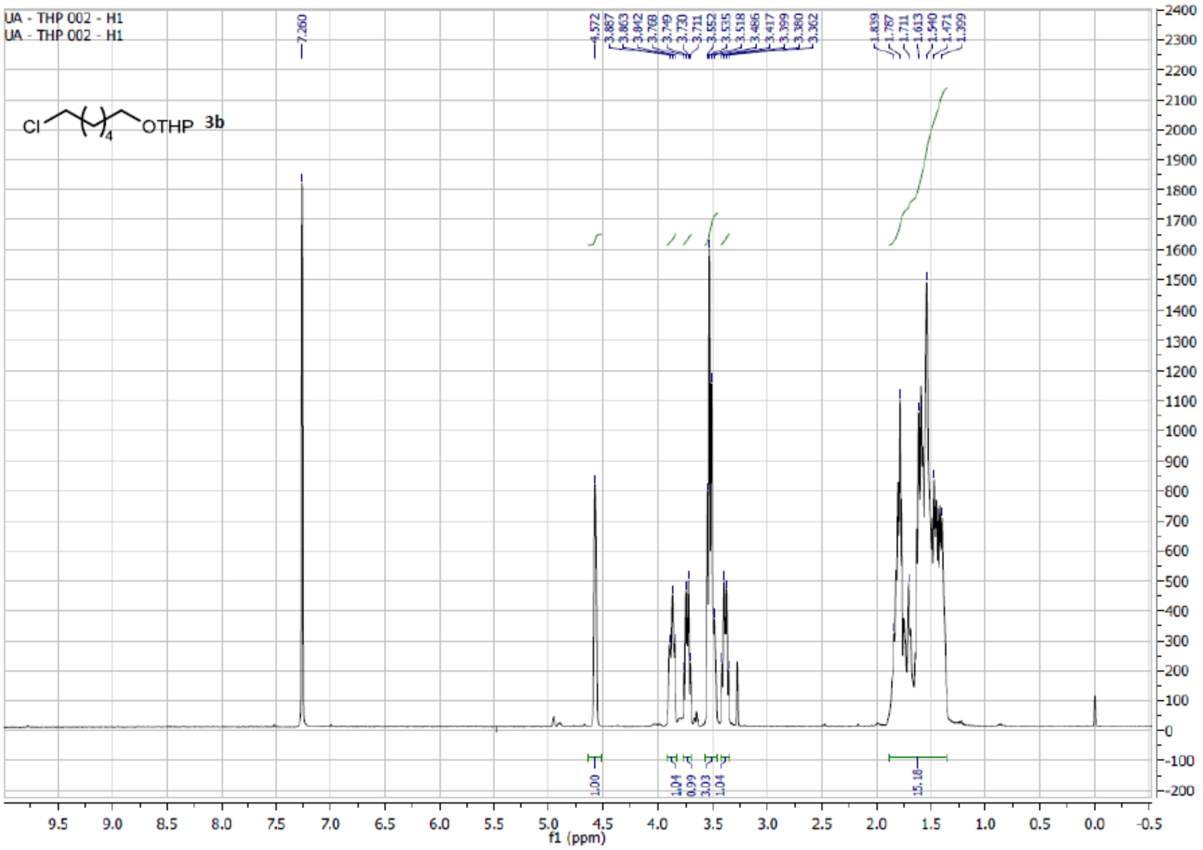
UA - THP 002 - H1
UA - THP 002 - H1
Cl OTHP 3b
f1 (ppm)

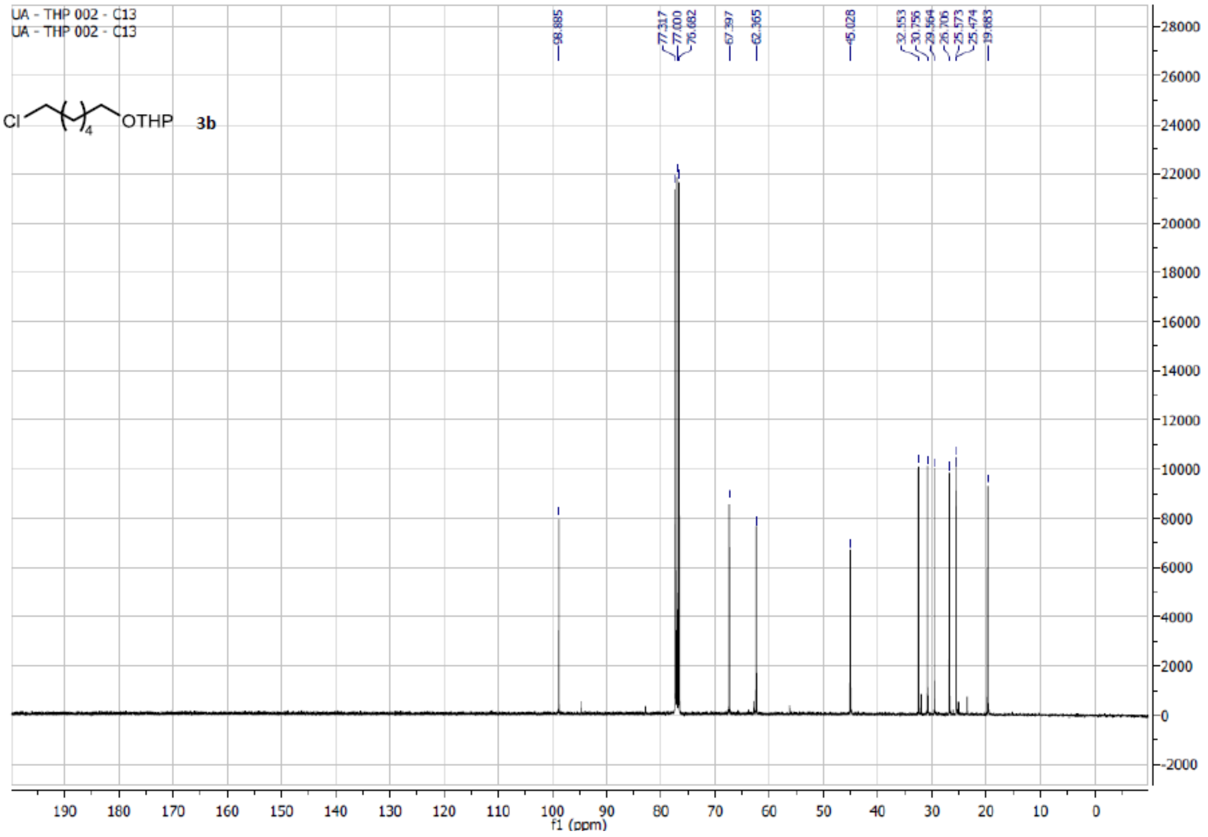
UA - THP 002 - C13
UA - THP 002 - C13
Cl OTHP 3b
f1 (ppm)

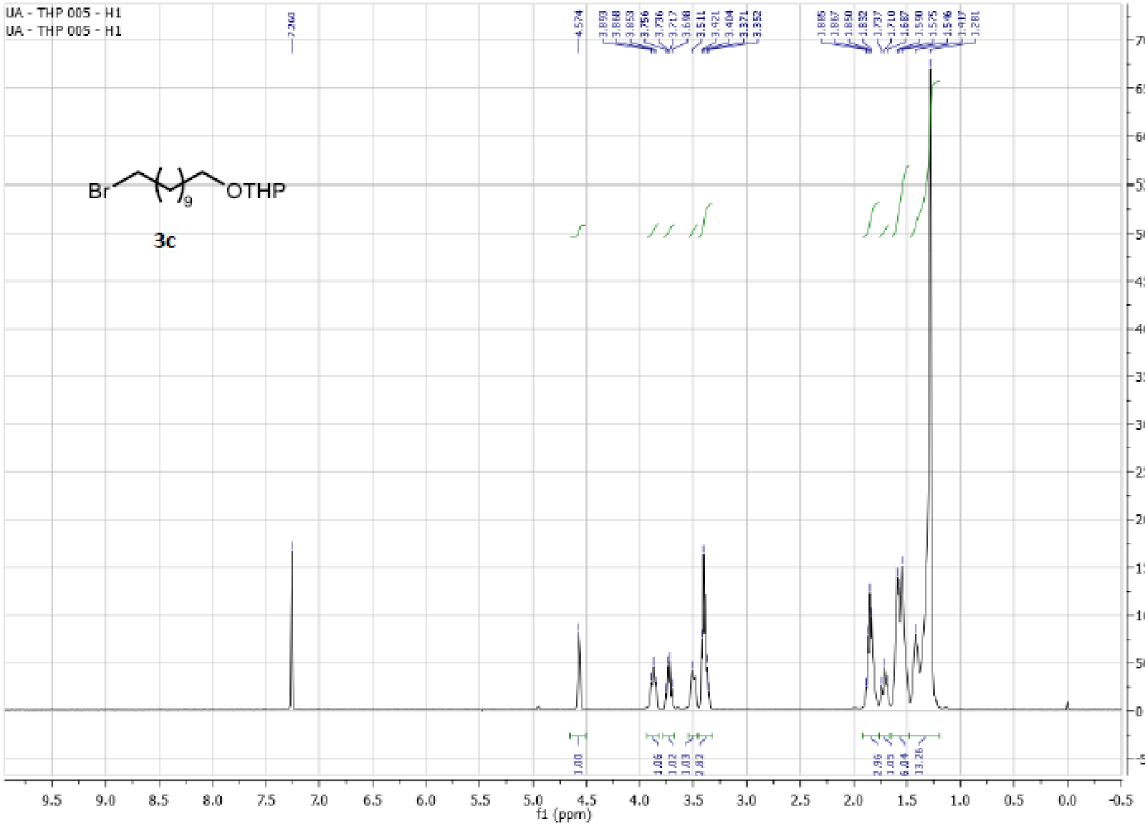
UA - THP 005 - H1
UA - THP 005 - H1
Br OTHP 9
3c
f1 (ppm)

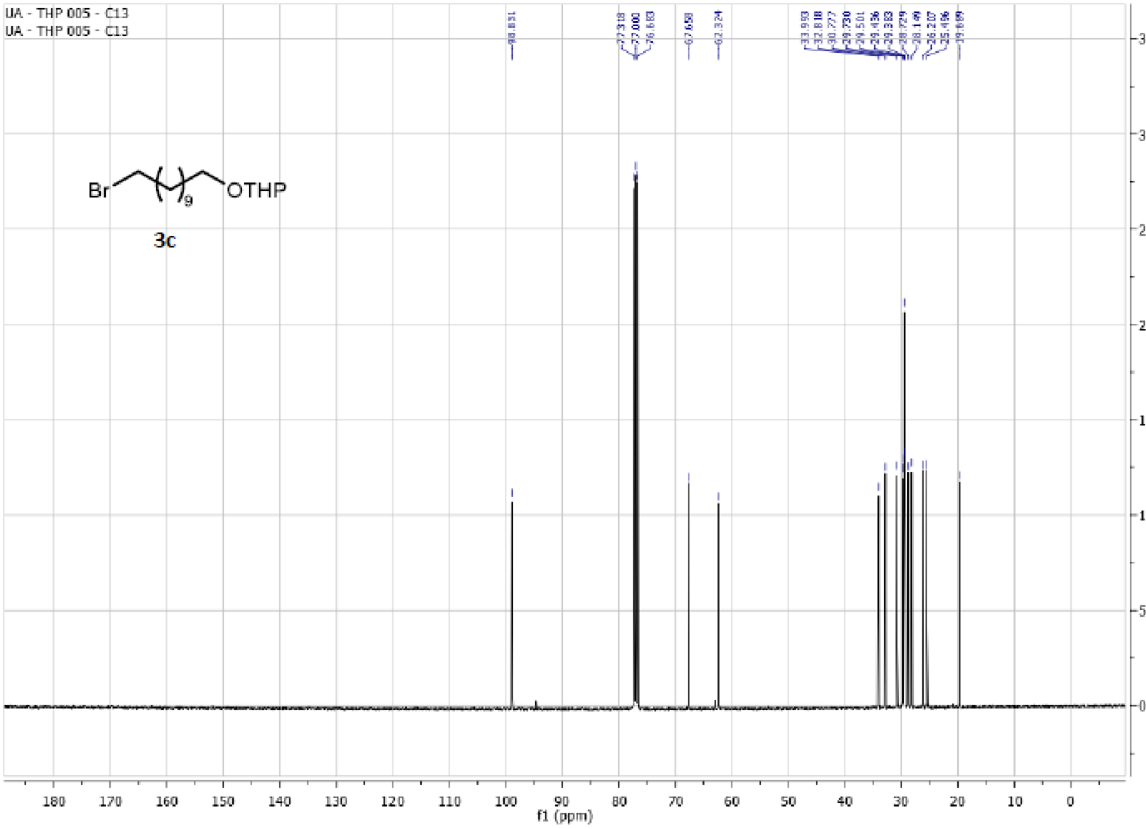
UA - THP 005 - C13
UA - THP 005 - C13
Br OTHP 9
3c
f1 (ppm)

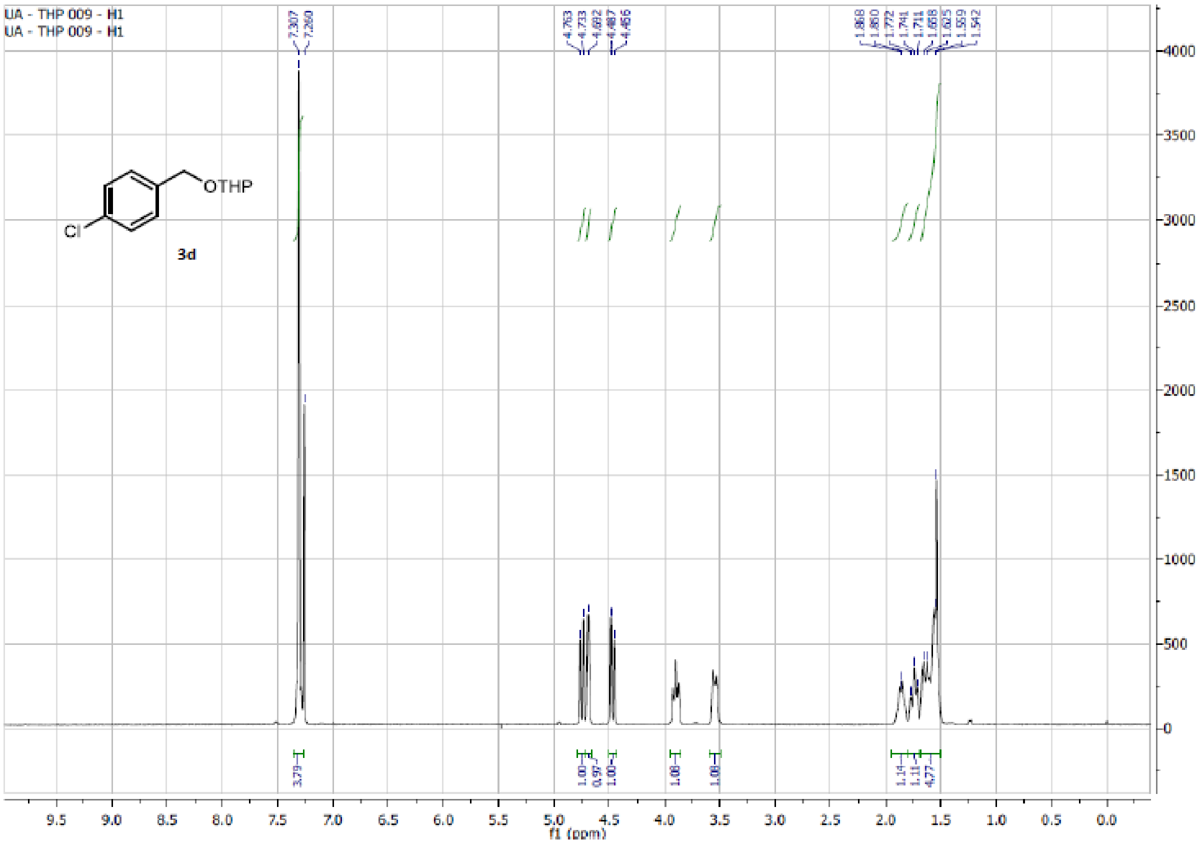
UA - THP 009 - H1
UA - THP 009 - H1
OTHP
Cl
3d
f1 (ppm)

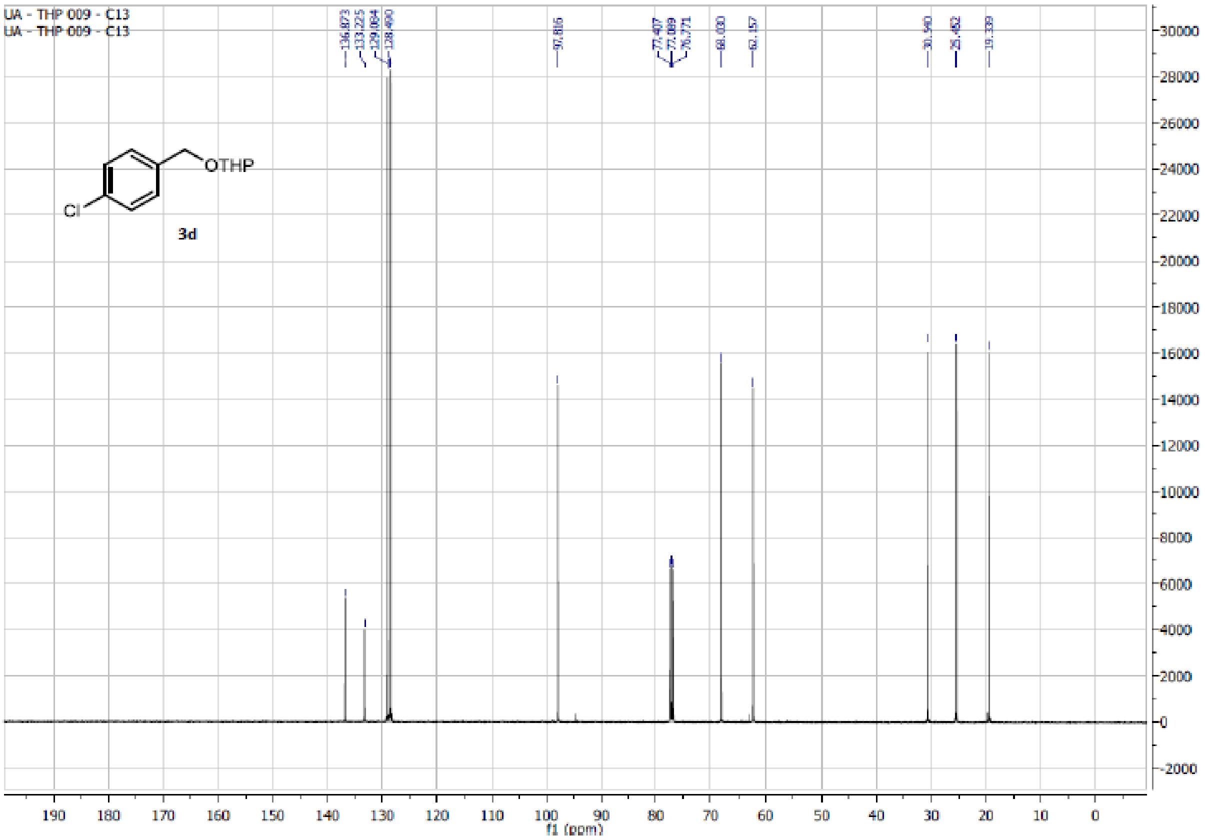
UA - THP 009 - C13
UA - THP 009 - C13
OTHP
Cl
3d
f1 (ppm)

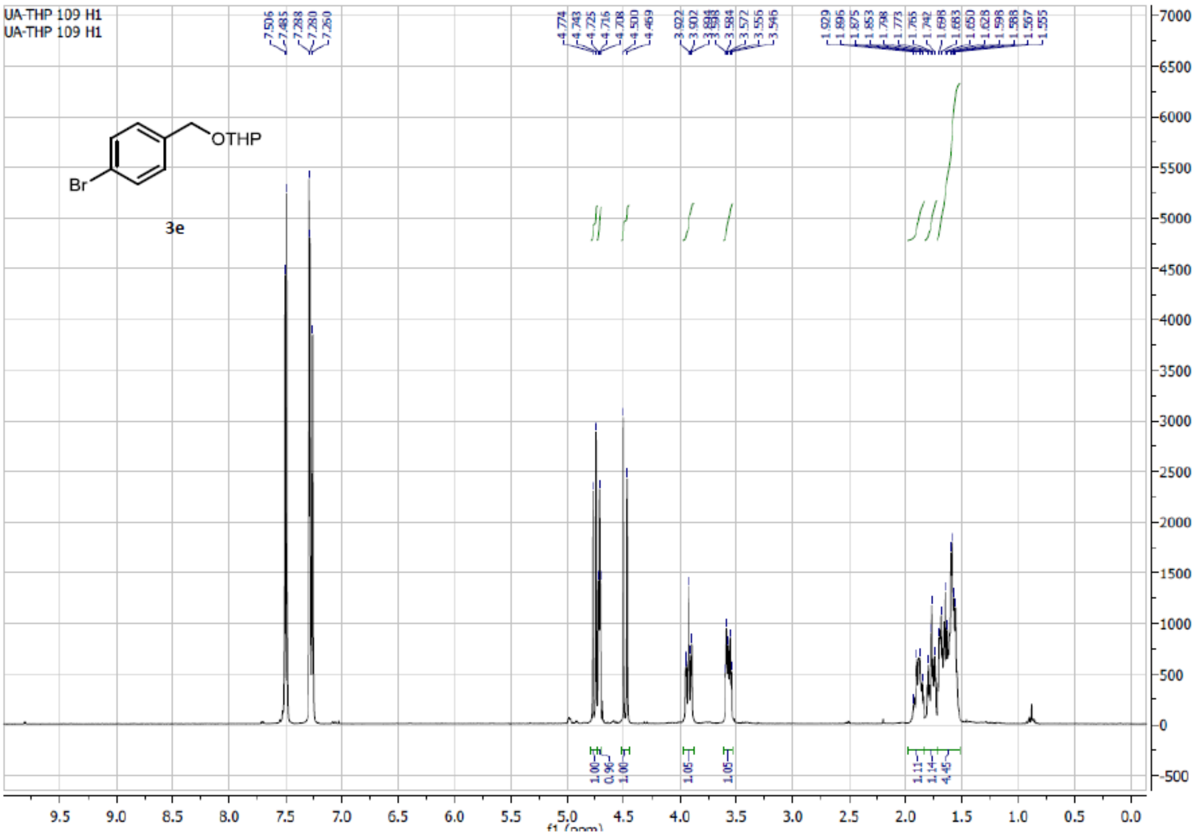
UA-THP 109 H1
UA-THP 109 H1
OTHP
Br
3e
f1 (ppm)

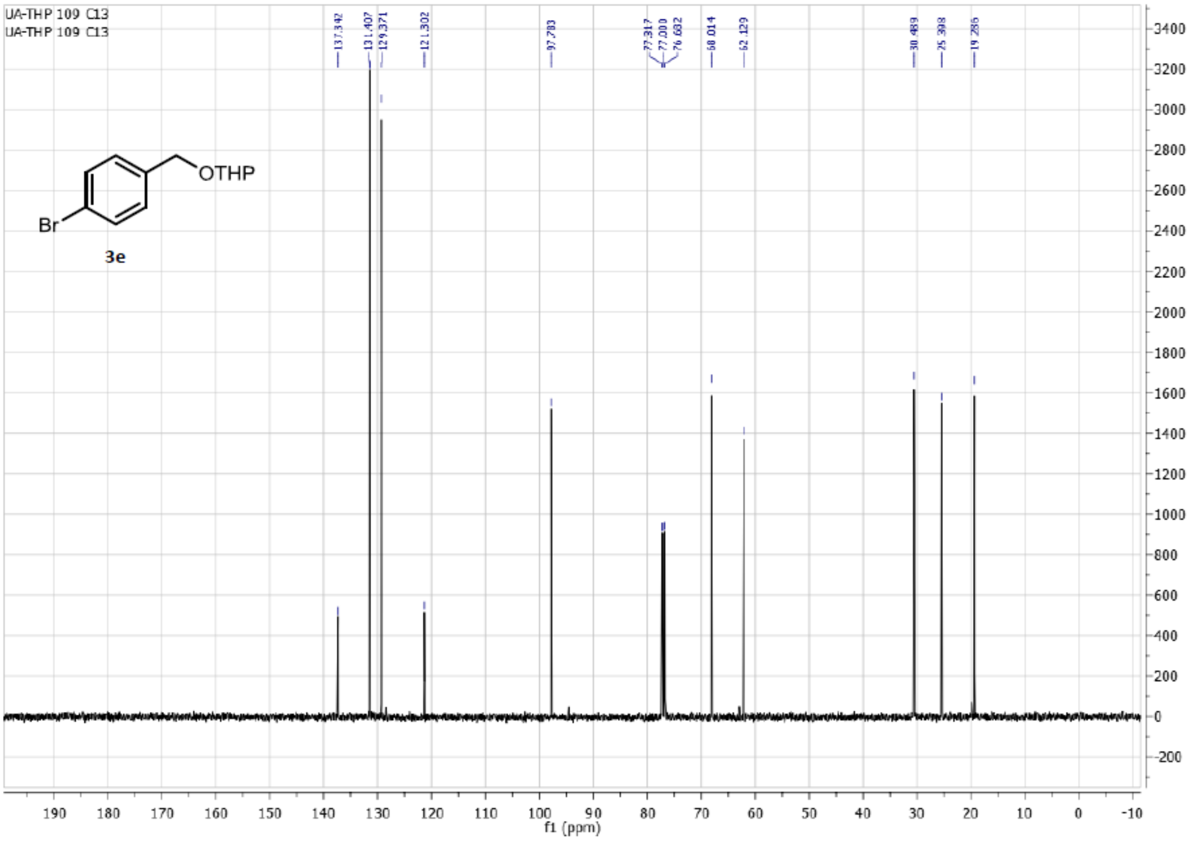
UA-THP 109 C13
UA-THP 109 C13
137.342
131.407
129.371
121.302
97.703
77.317
77.000
76.682
68.014
62.129
30.489
25.398
19.286
OTHP
Br
3e
f1 (ppm)

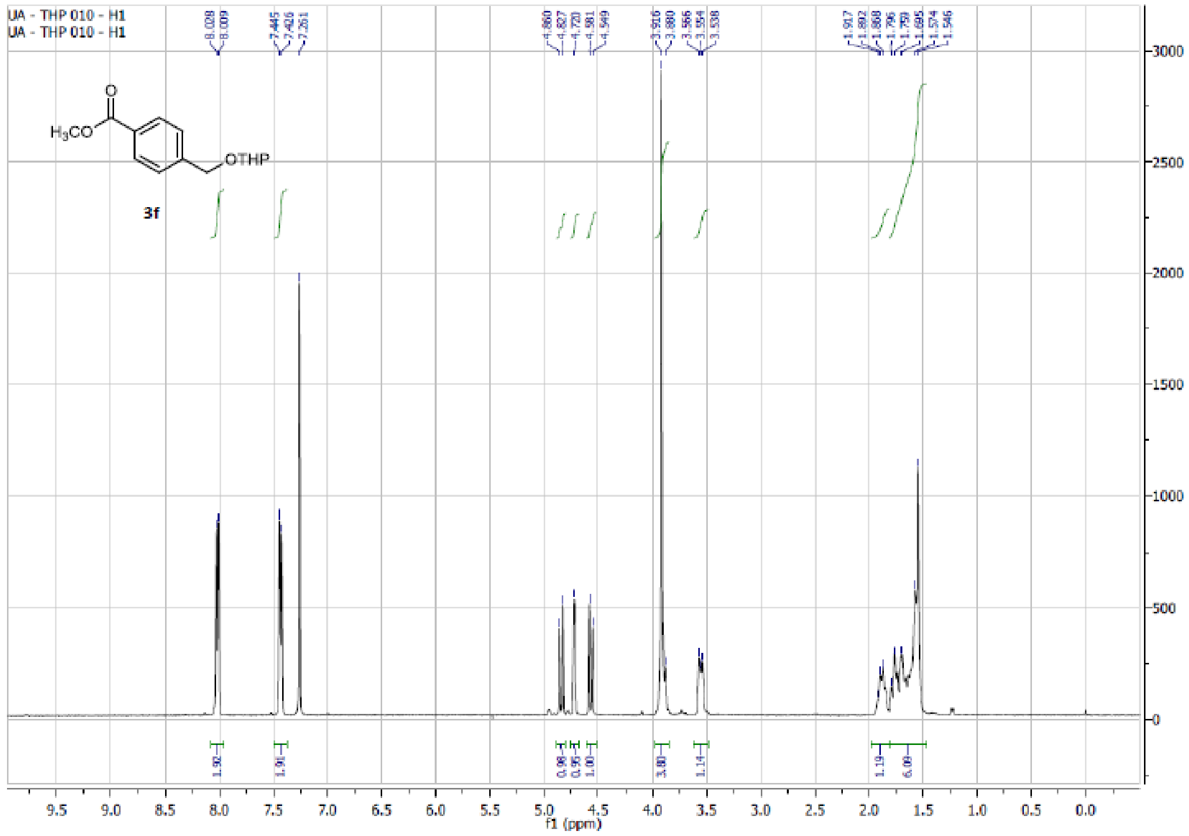
UA - THP 010 - H1
UA - THP 010 - H1
H3CO
OTHP
3f
f1 (ppm)

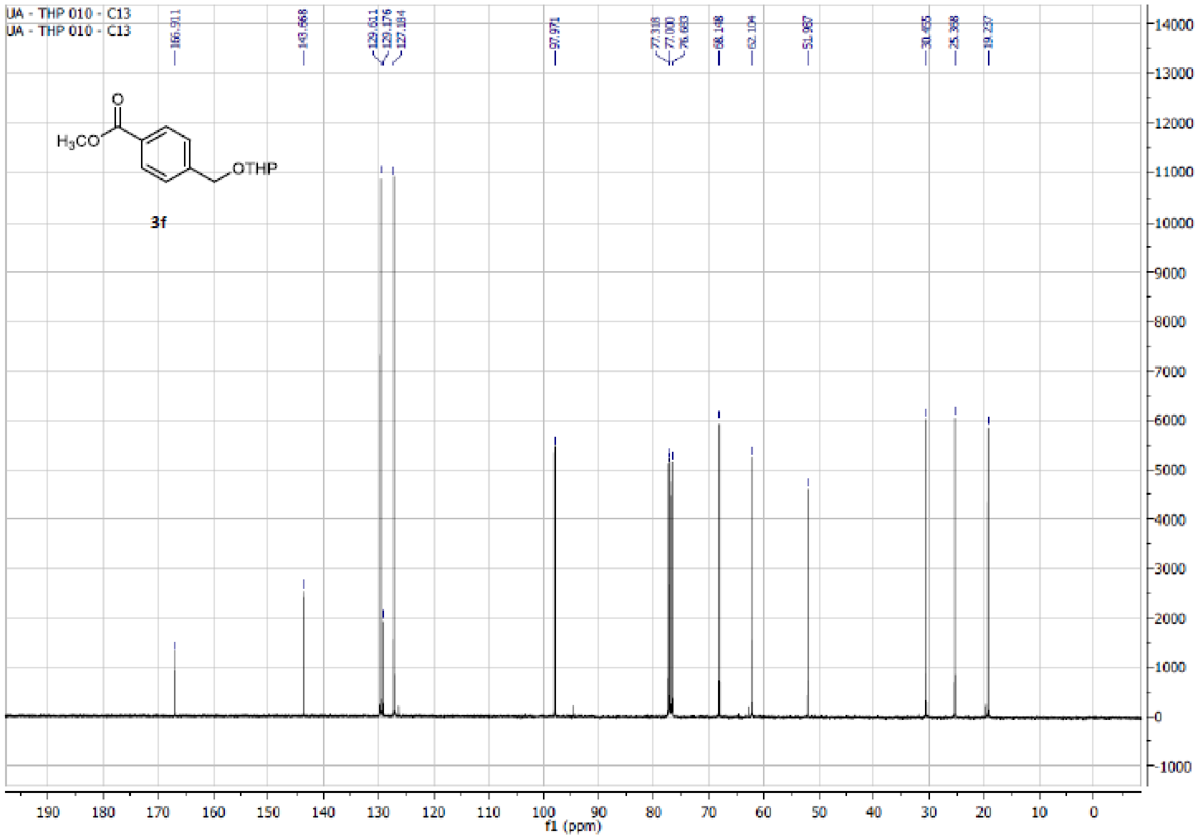
UA - THP 010 - C13
UA - THP 010 - C13
H3CO
OTHP
3f
f1 (ppm)

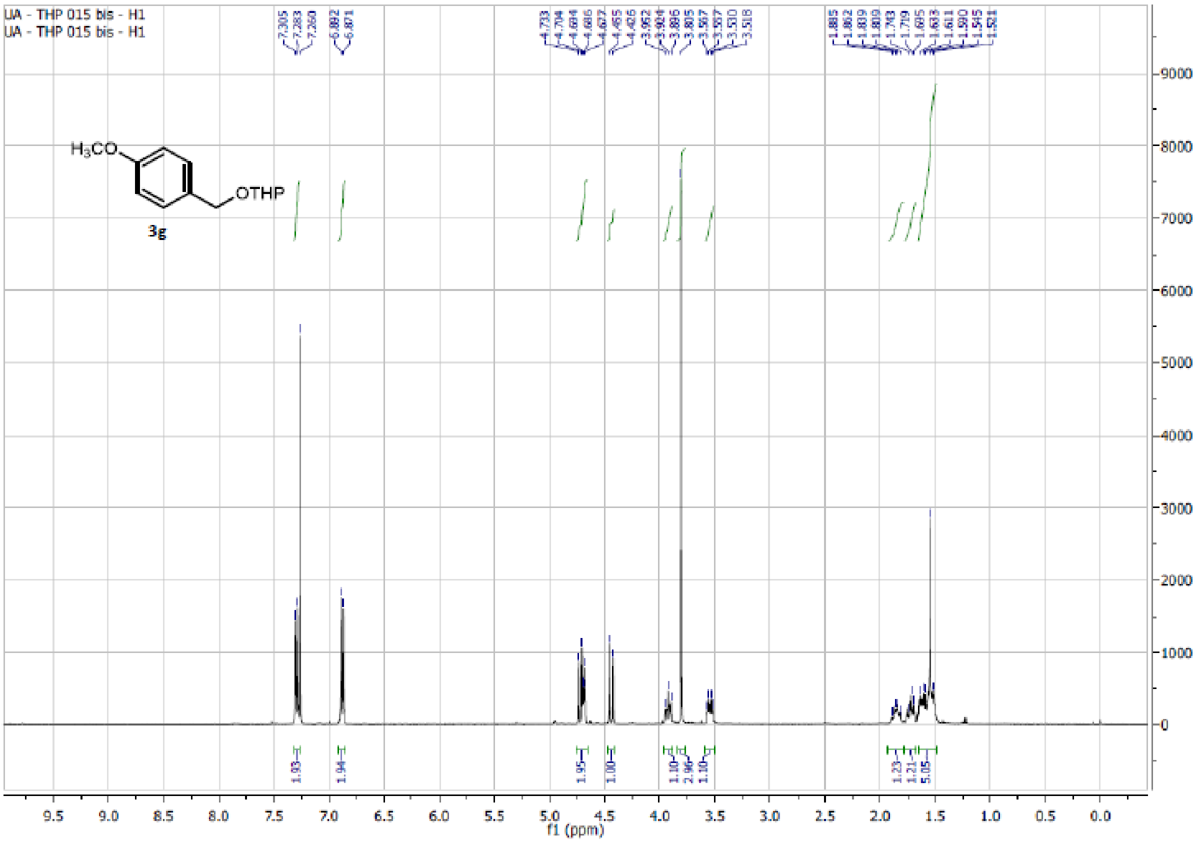
UA - THP 015 bis - H1
UA - THP 015 bis - H1
H3CO
OTHP
3g
f1 (ppm)

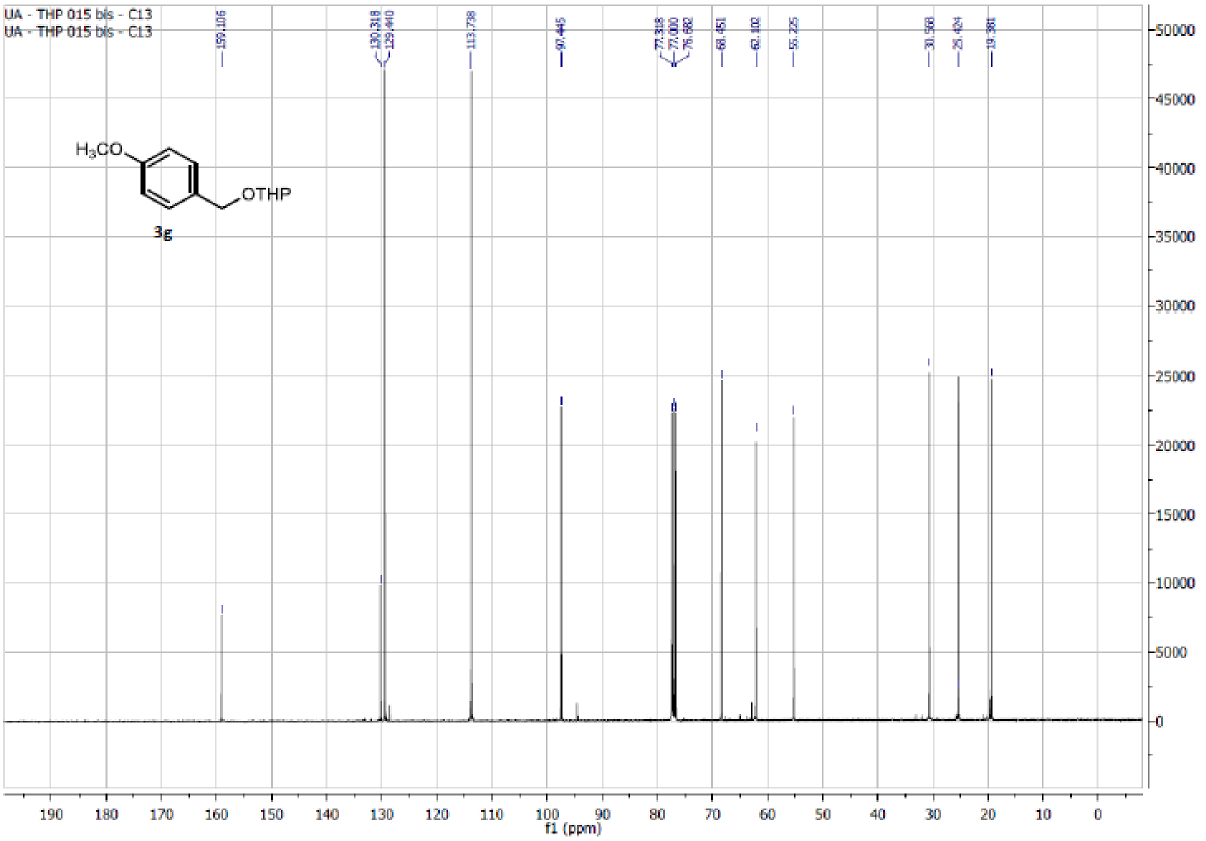
UA - THP 015 bis - C13
UA - THP 015 bis - C13
H3CO
OTHP
3g
f1 (ppm)

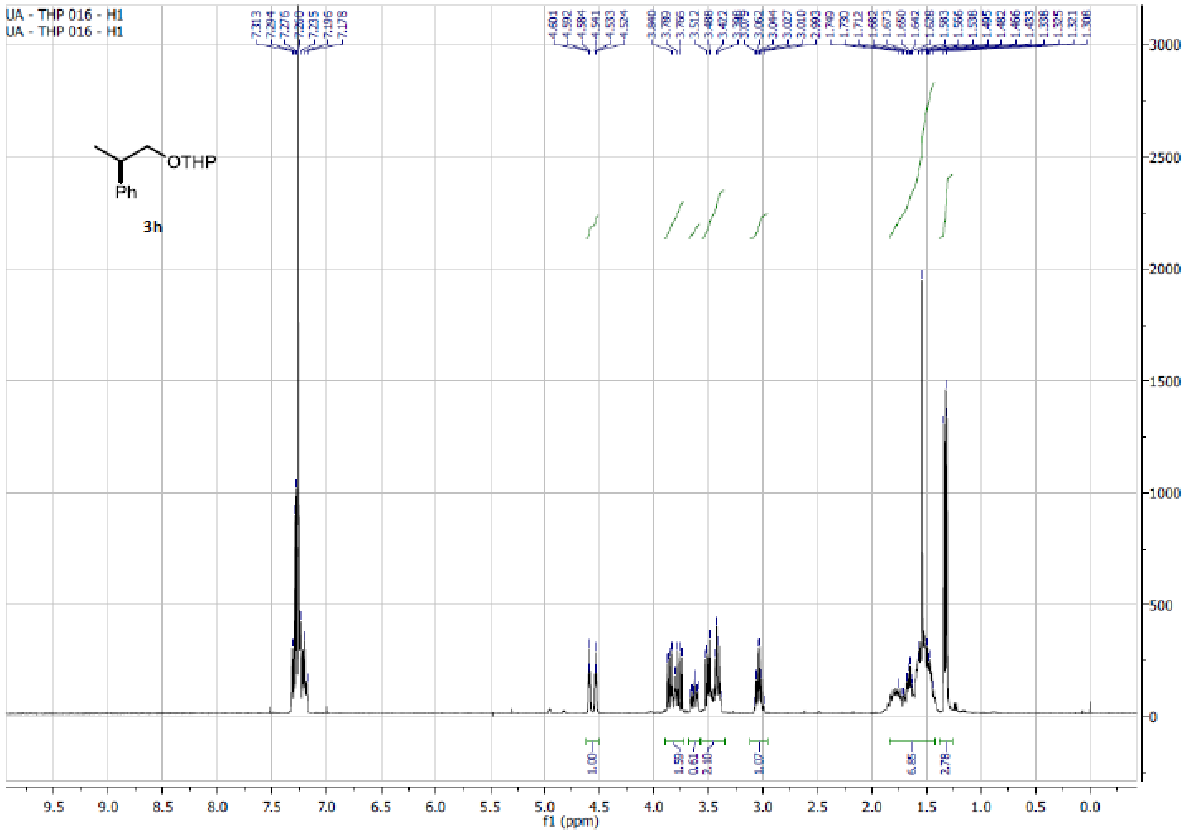
UA - THP 016 - H1
UA - THP 016 - H1
OTHP
Ph
3h
f1 (ppm)

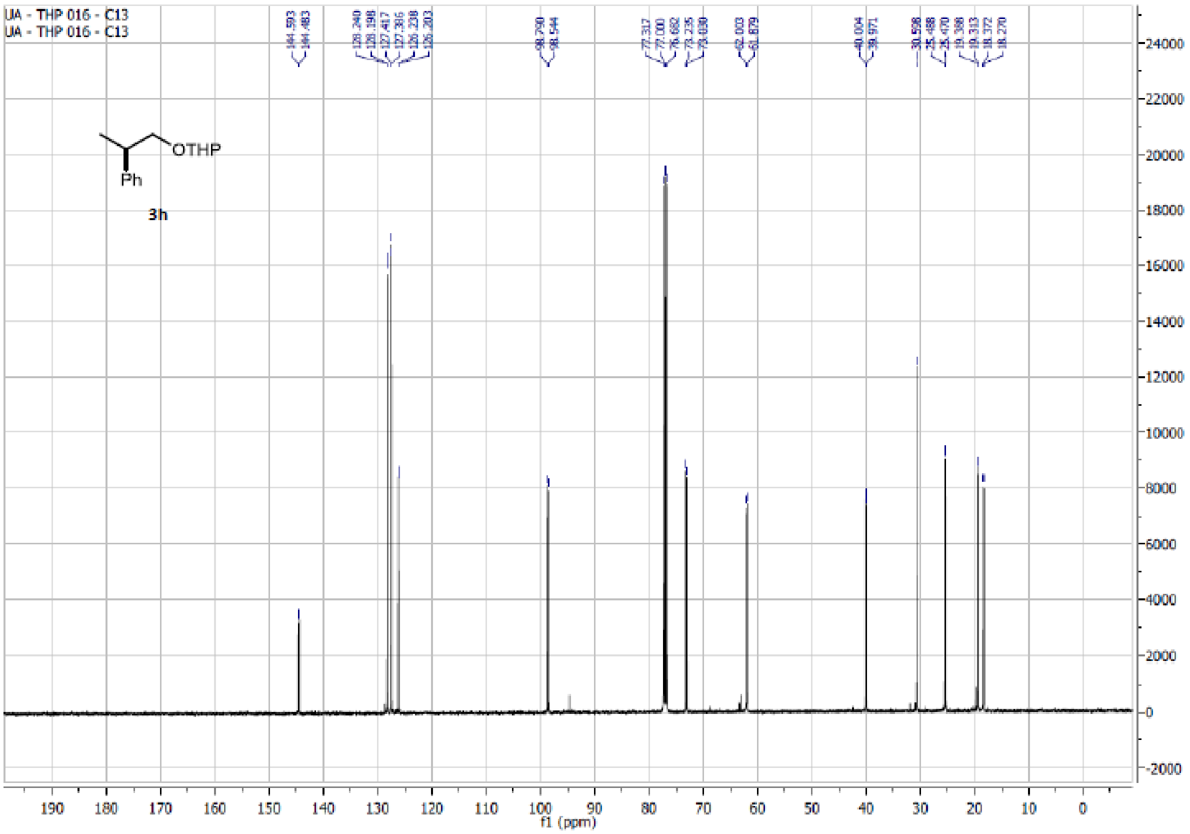
UA - THP 016 - C13
UA - THP 016 - C13
OTHP
Ph
3h
f1 (ppm)

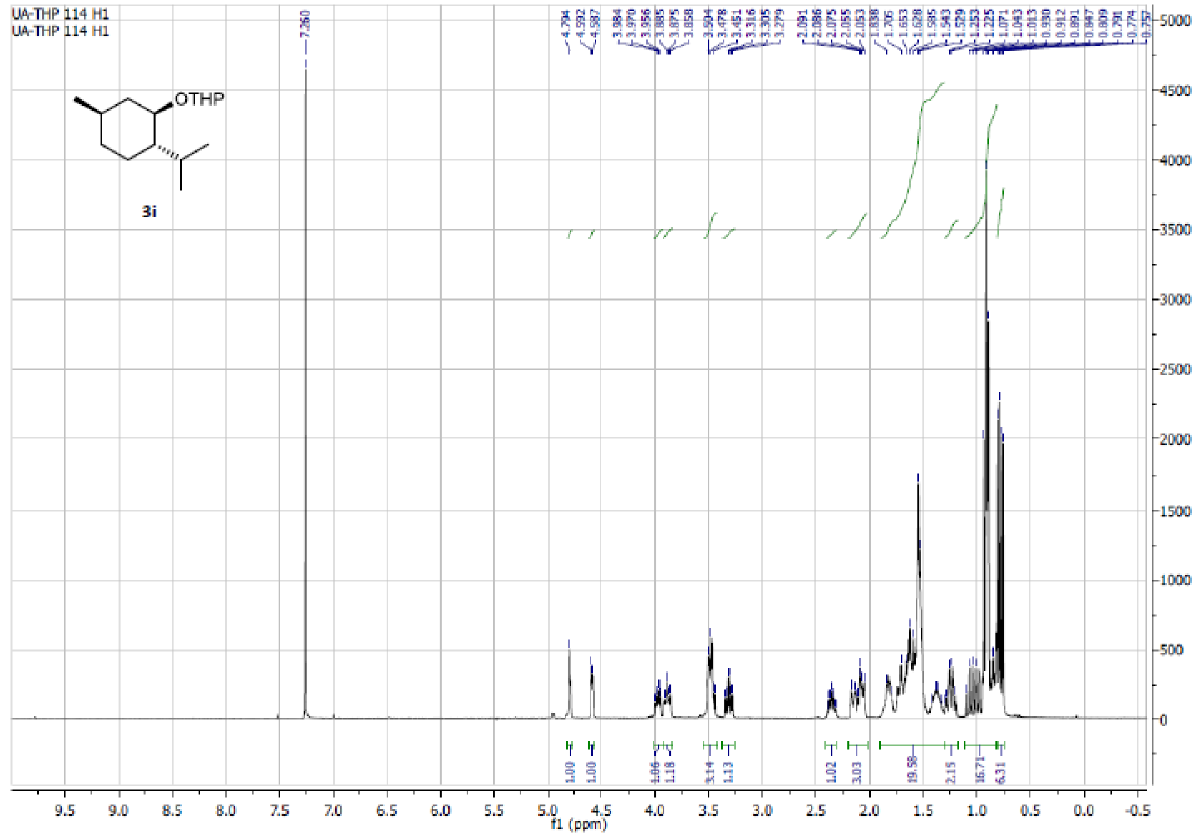

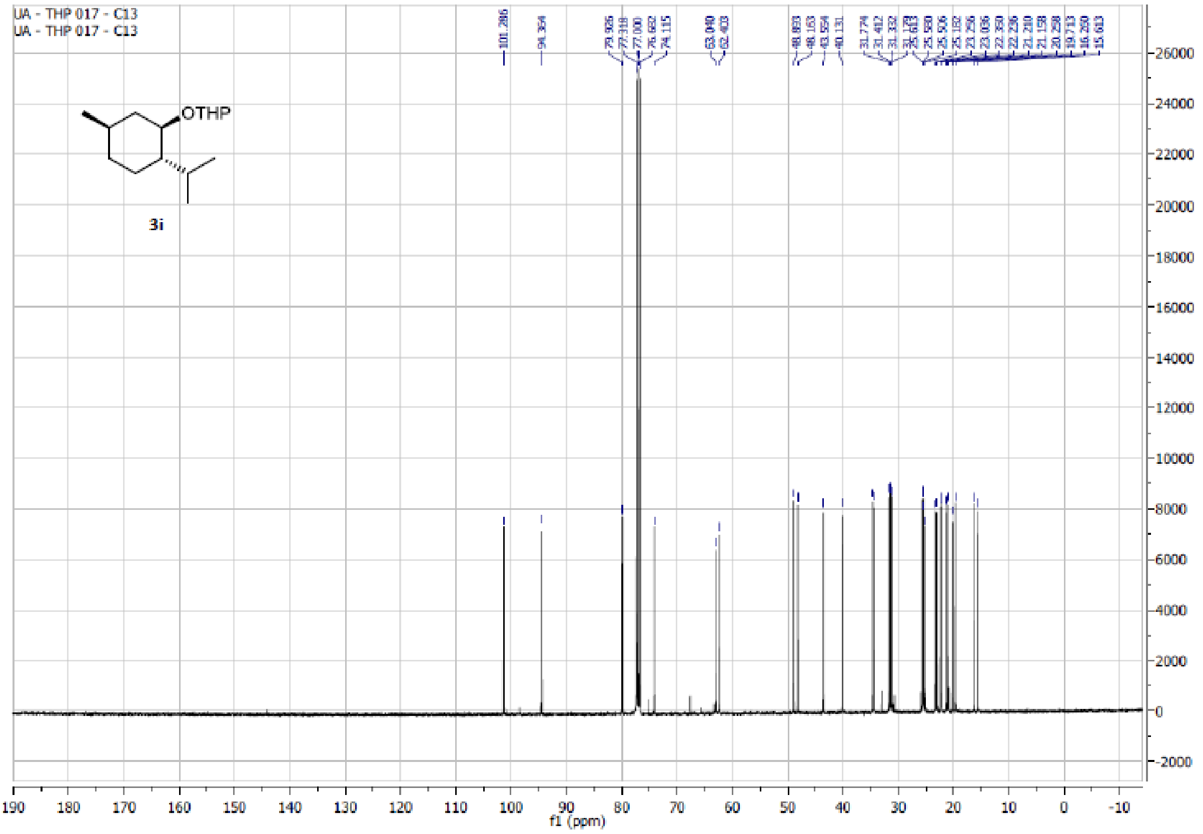

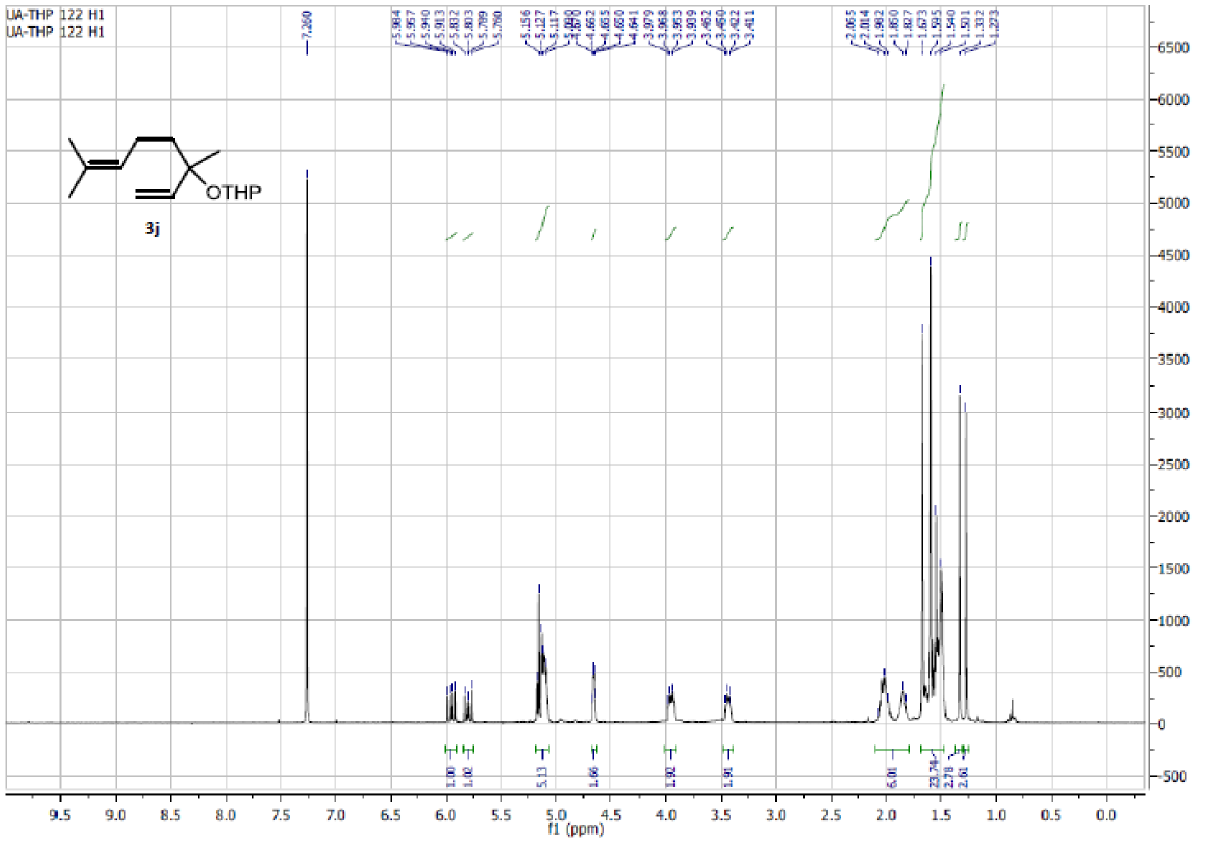
UA-THP 122 H1
UA-THP 122 H1
OTHP
3j
f1 (ppm)

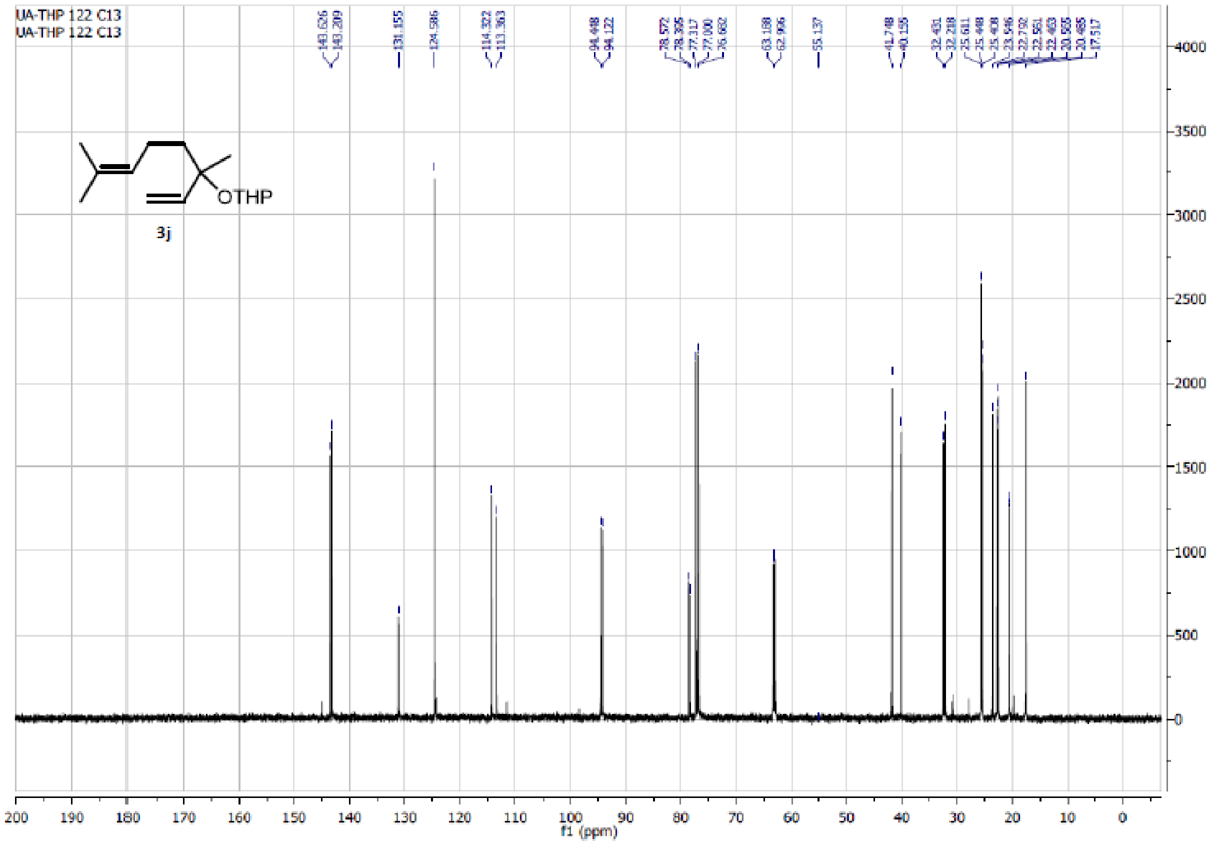
UA-THP 122 C13
UA-THP 122 C13
OTHP
3j
f1 (ppm)

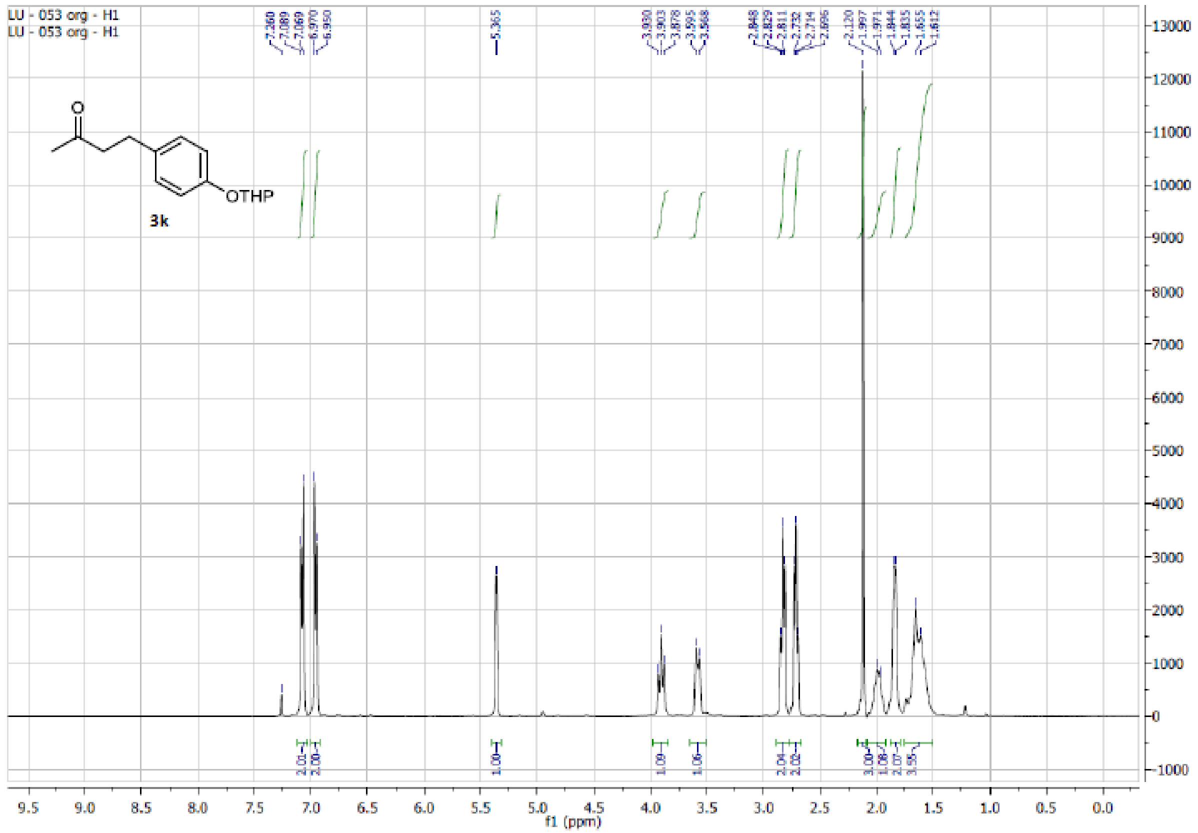
LU - 053 org - H1
LU - 053 org - H1
7.260
7.089
7.069
6.970
6.950
5.365
3.930
3.903
3.878
3.595
3.568
2.848
2.829
2.811
2.732
2.714
2.696
2.120
1.997
1.971
1.844
1.835
1.655
1.612
O
OTHP
3k
2.01
2.00
1.00
1.09
1.06
2.04
2.02
3.00
1.08
2.07
3.55
f1 (ppm)

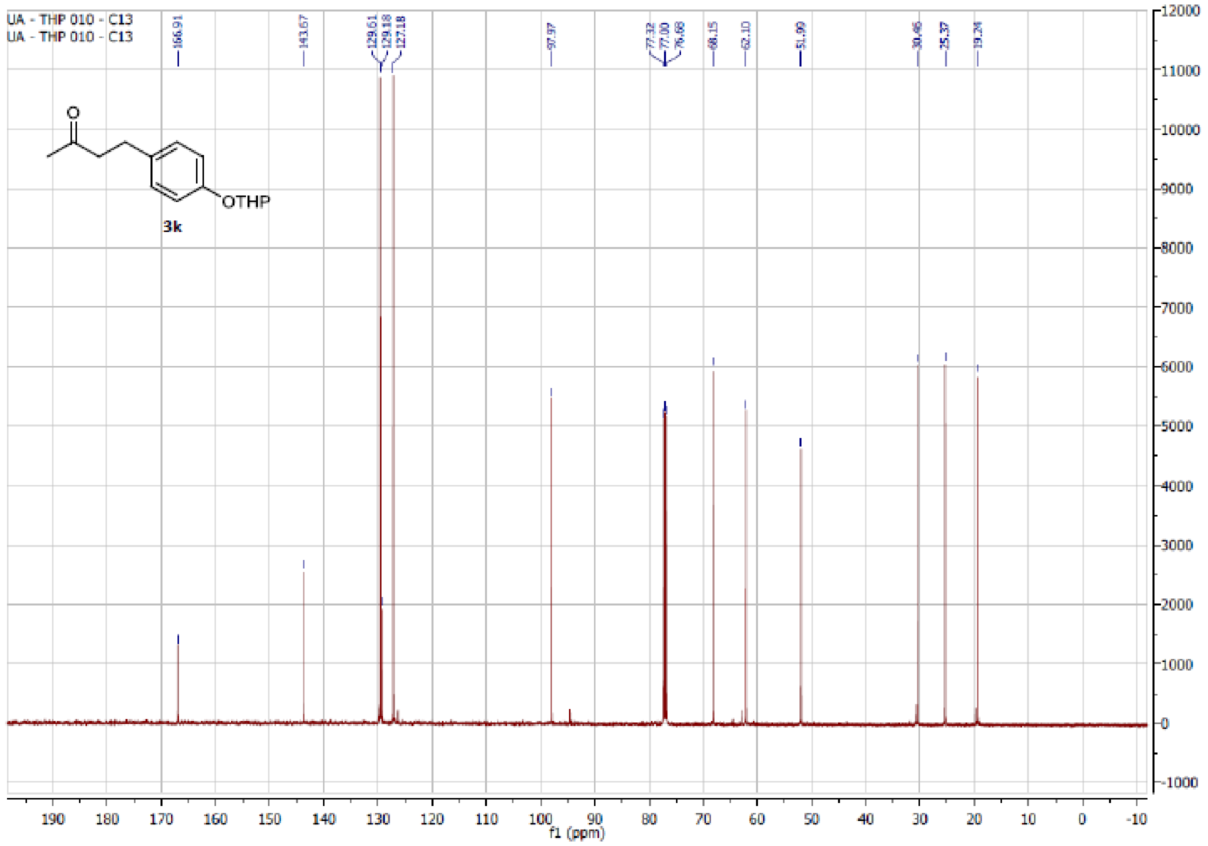
UA - THP 010 - C13
UA - THP 010 - C13
166.91
143.67
129.61
129.18
127.18
97.97
77.32
77.00
76.68
68.15
62.10
51.99
30.46
25.37
19.24
O
OTHP
3k
f1 (ppm)

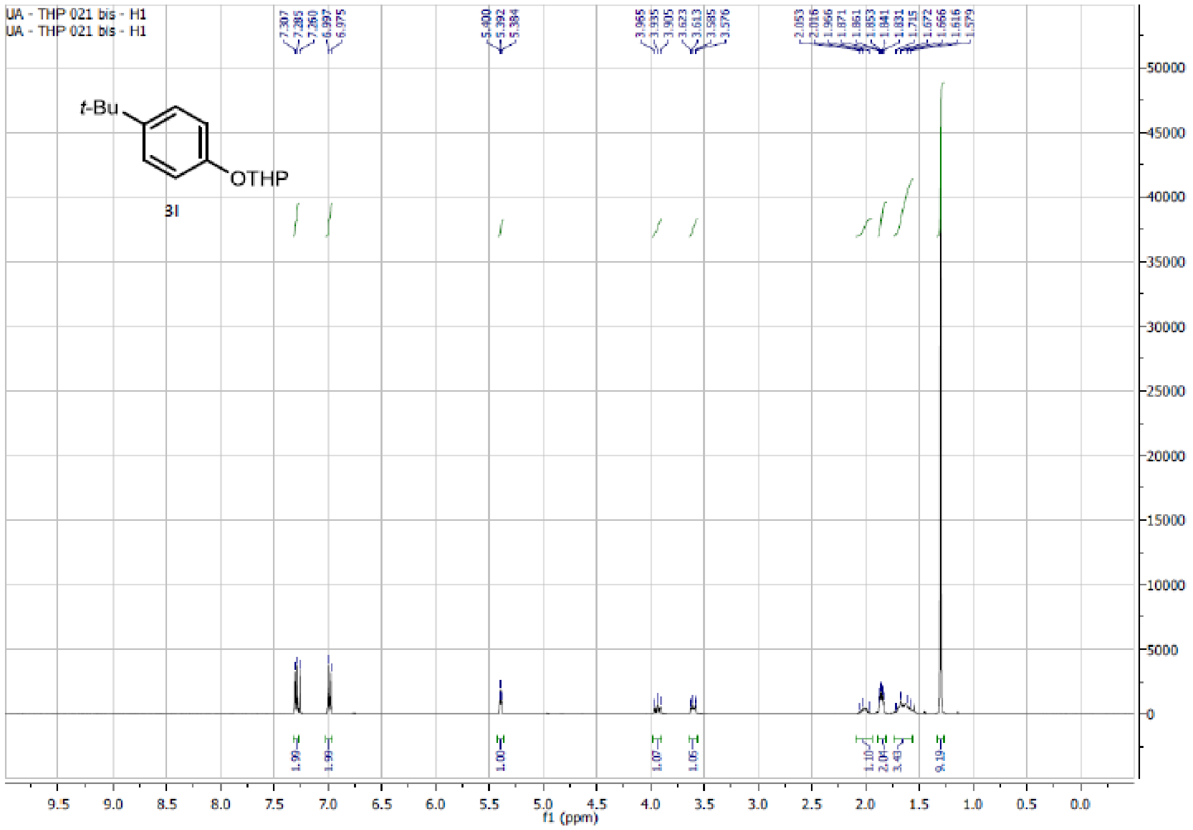

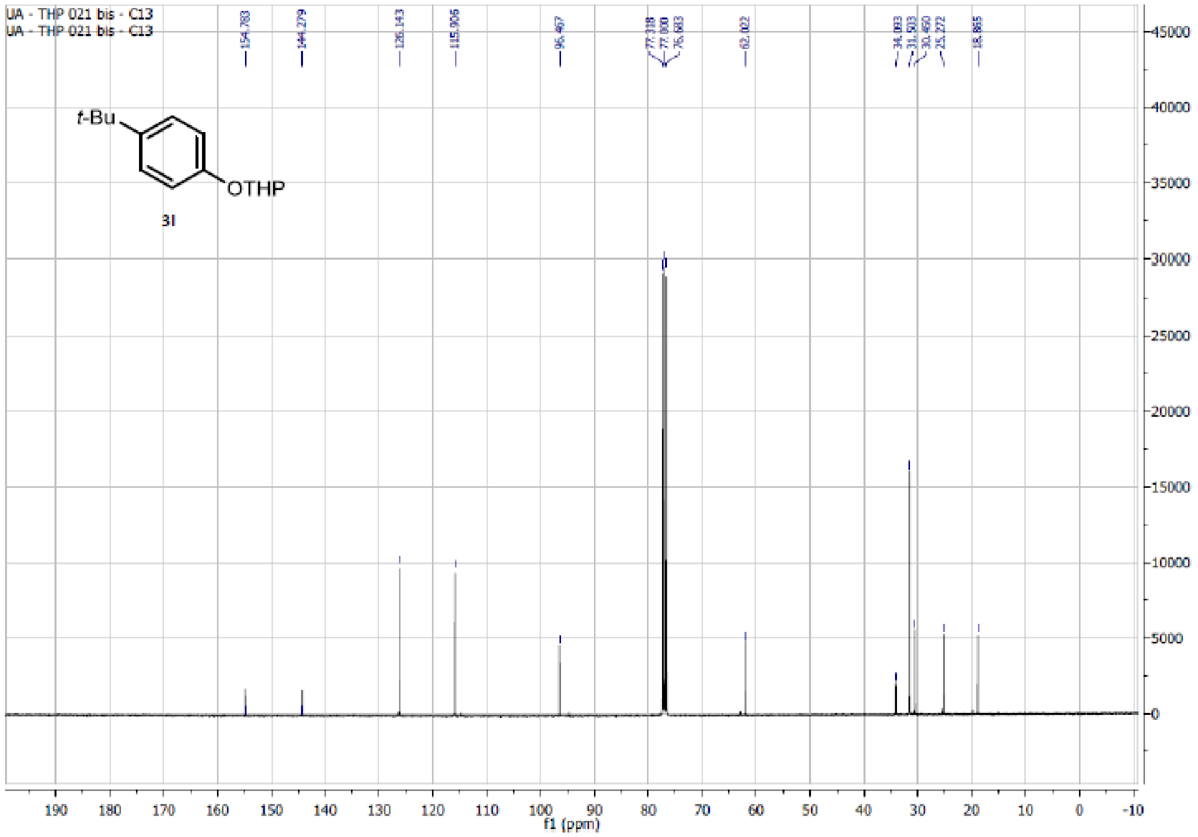

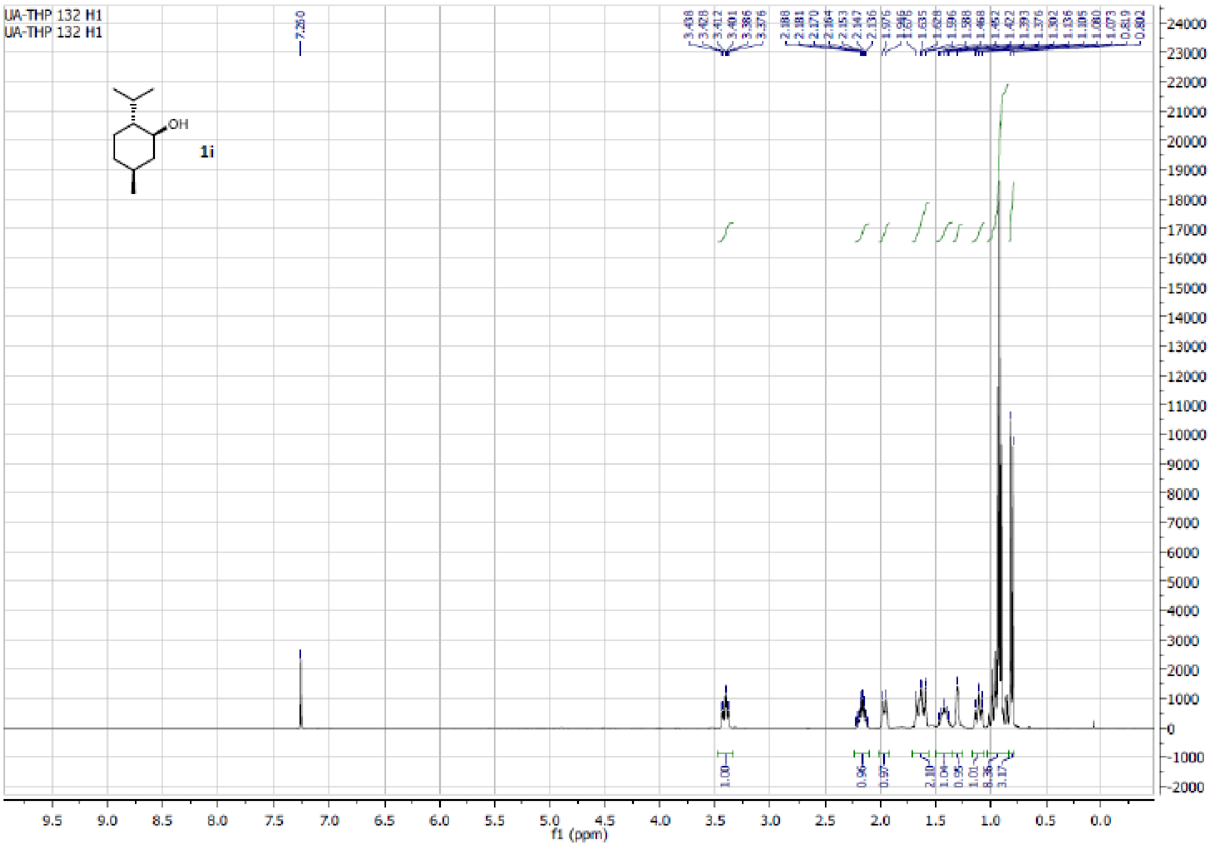
UA-THP 132 H1
UA-THP 132 H1
OH
1i
f1 (ppm)

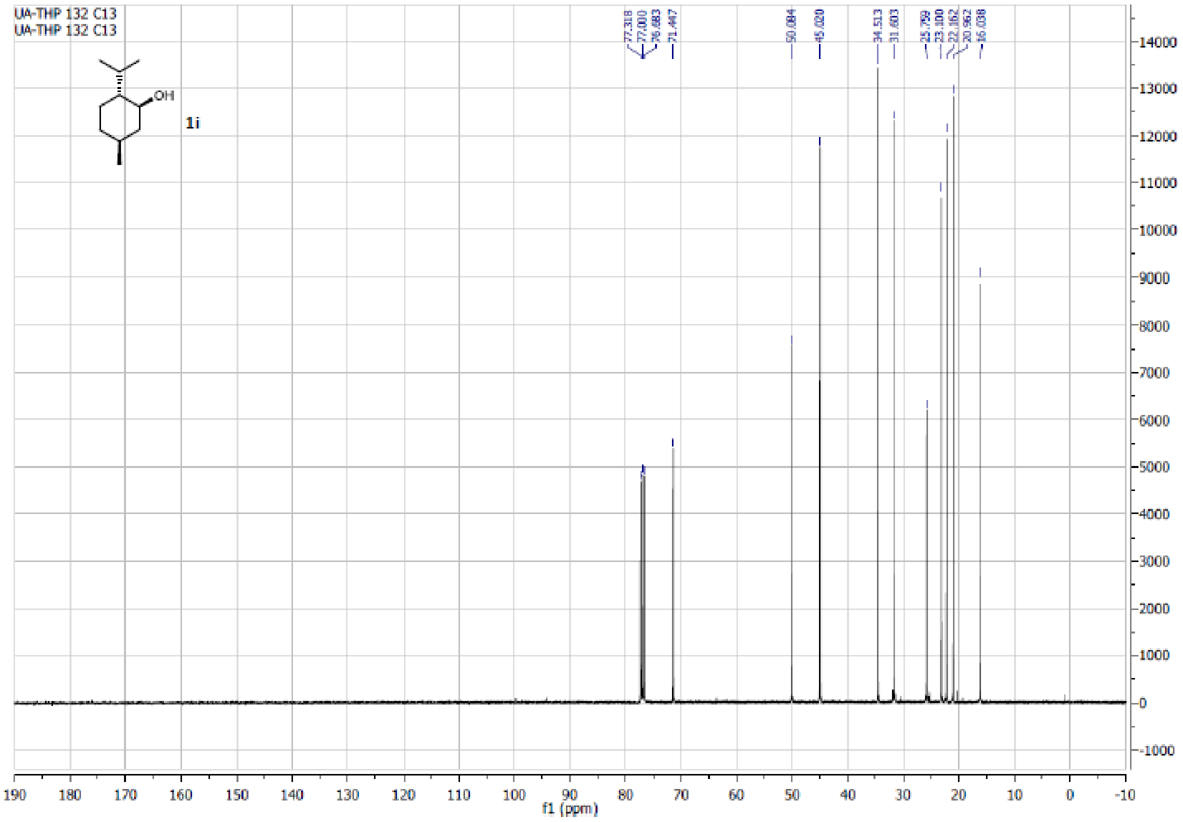
UA-THP 132 C13
UA-THP 132 C13
OH
1i
77.318
77.000
76.683
71.447
50.084
45.020
34.513
31.603
25.759
23.100
22.162
20.962
16.038
f1 (ppm)

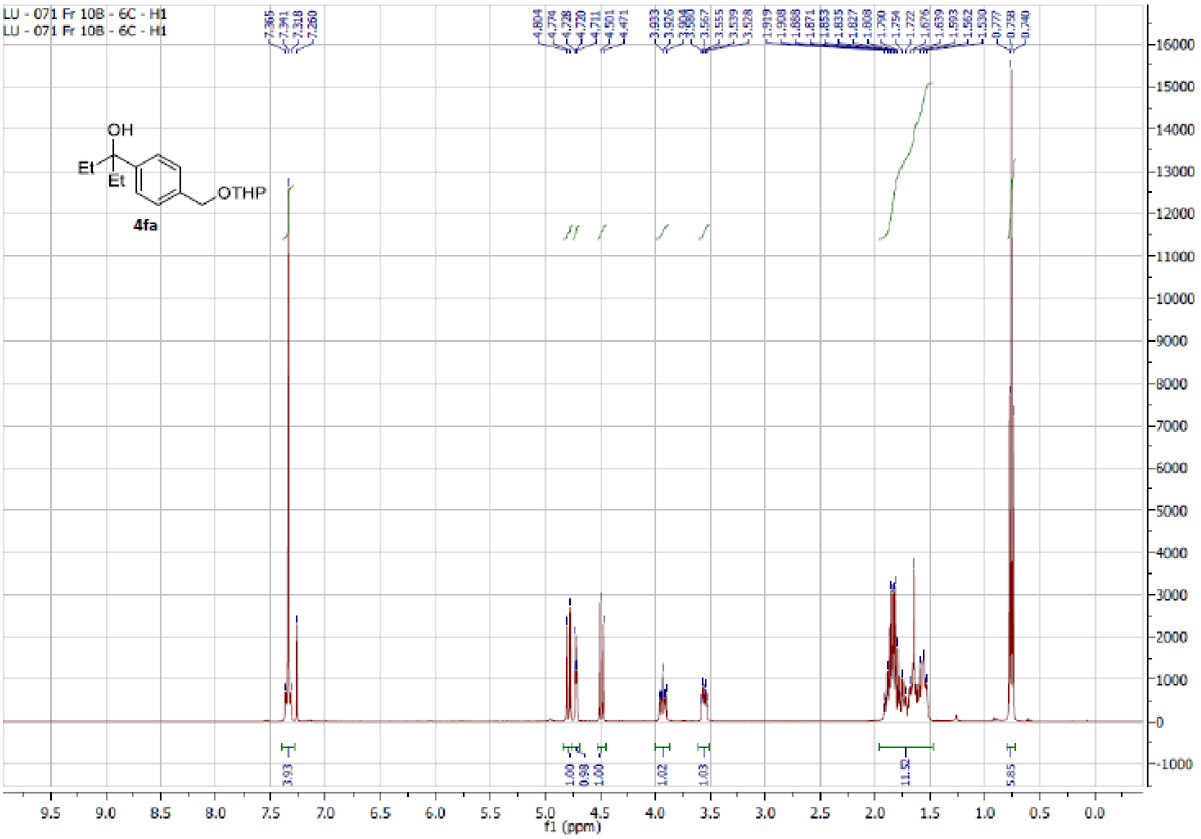
LU - 071 Fr 10B - 6C - H1
LU - 071 Fr 10B - 6C - H1
OH
Et
Et
OTHP
4fa
f1 (ppm)

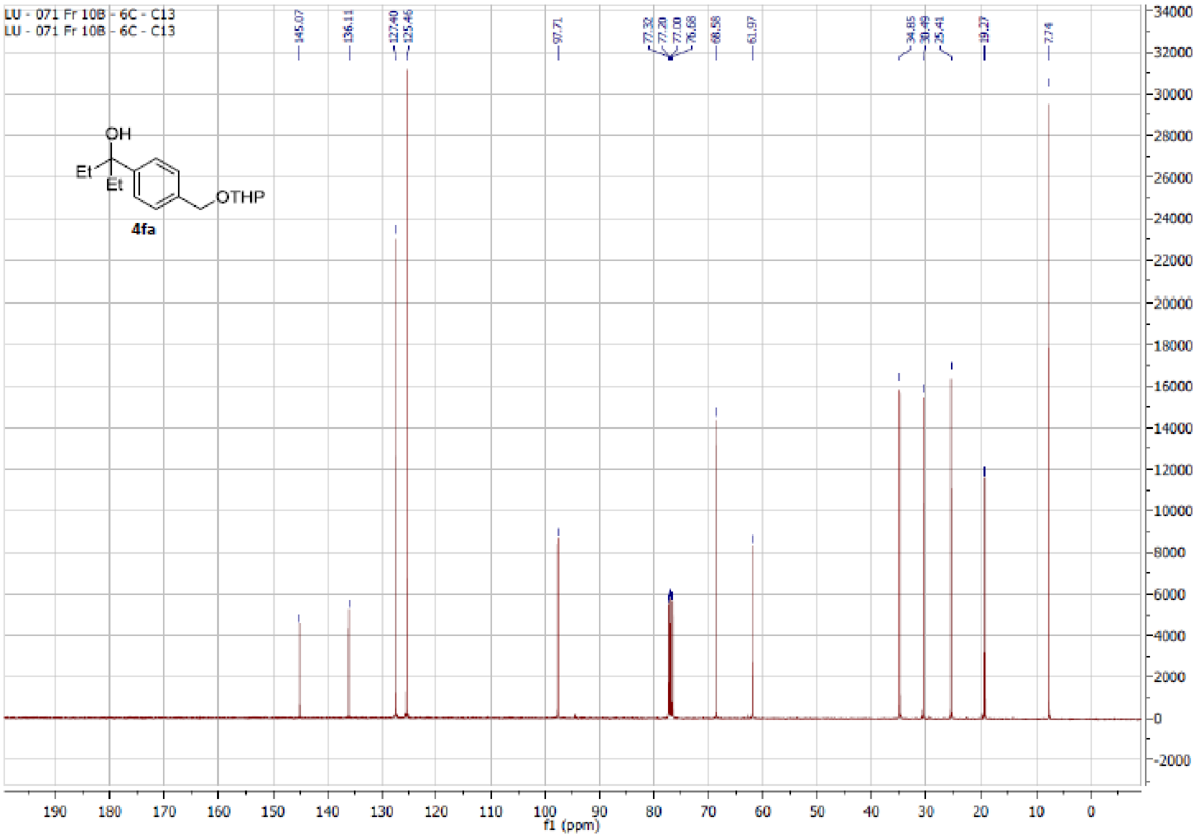
LU - 071 Fr 10B - 6C - C13
LU - 071 Fr 10B - 6C - C13
145.07
136.11
127.40
125.46
97.71
77.32
77.20
77.00
76.68
68.58
61.97
34.85
30.49
25.41
19.27
7.74
OH
Et
Et
OTHP
4fa
f1 (ppm)

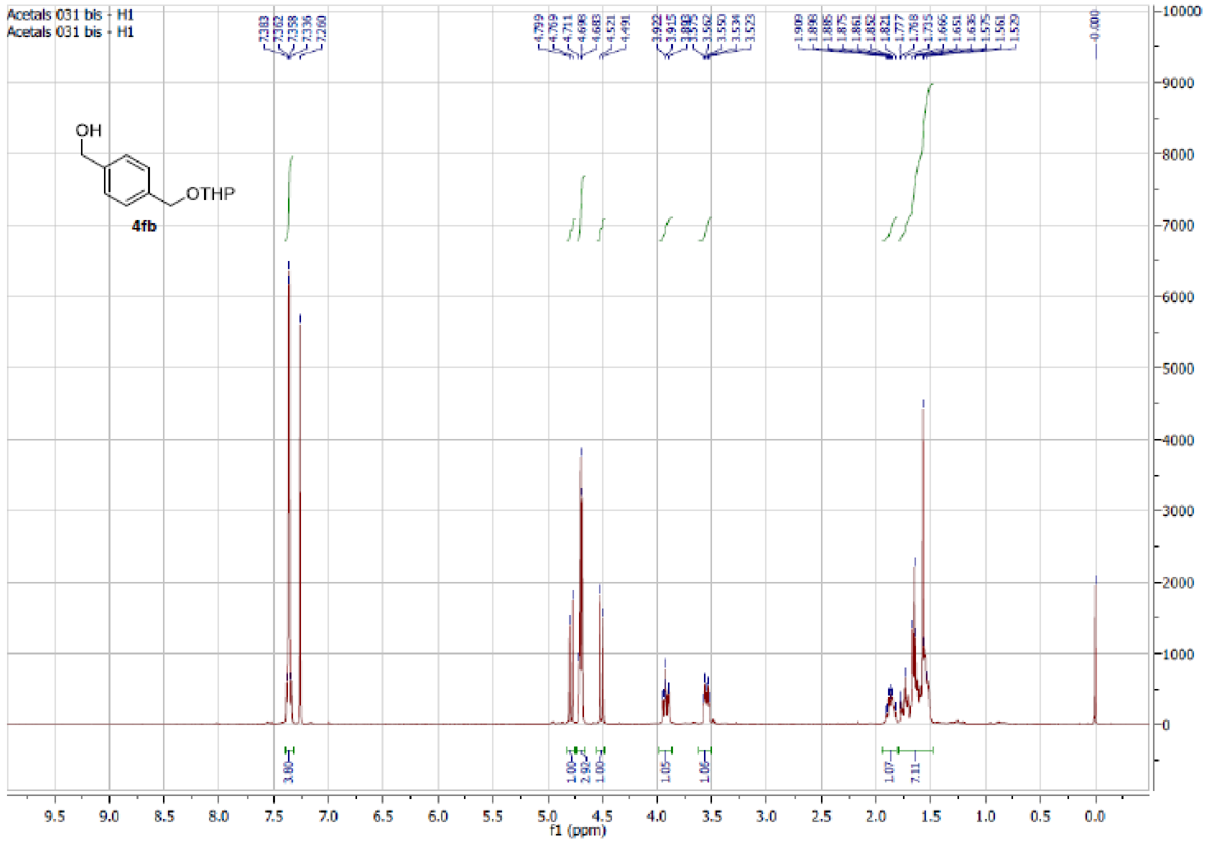

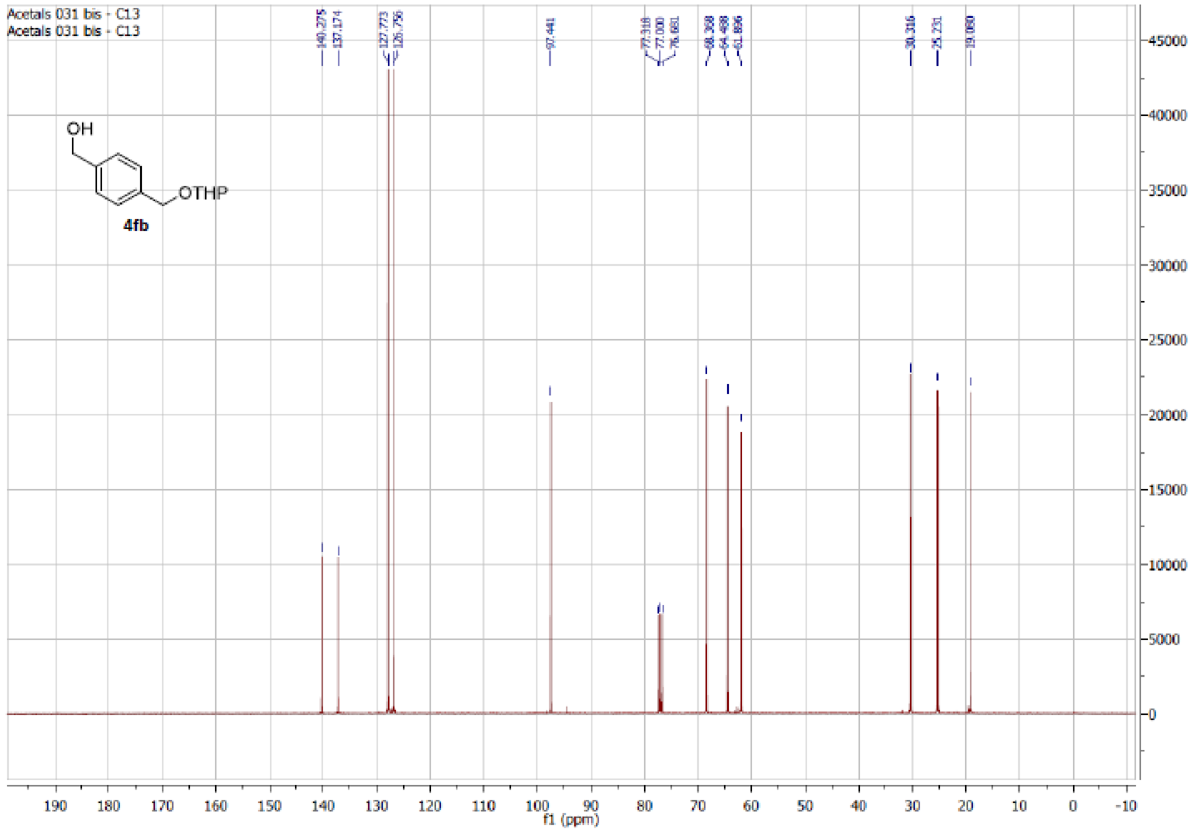

References

1. Riego, J. M.; Sedin, Z.; Zaldívar, J. M. ; Marziano, N. C.; Tortato ; C. *Tetrahedron Lett.*, **1996**, 513-516. doi: 10.1016/0040-4039(95)02174-4.

2. Kumar, B.; Aga, M. A.; Mukherjee, D.; Chimni, S. S.; Taneja, S. C. *Tetrahedron Lett.* **2009**, *50*, 6236-6240. doi: 10.1016/j.tetlet.2009.09.026.

3. Lee, B. Y.; Oh, J. S. *Macromolecules* **2000**, *33*, 3194-3195. doi: 10.1021/ma9920386.

4. Fournet, A.; Achard, R.; Morel, J. *Compt. Rend.* **1965**, *260*, 5054-5044.

5. Banaszak, E.; Xu, L.-W.; Bardeau, J.-F.; Castanet, A.-S.; Mortier, J. *Tetrahedron* **2009**, *65*, 3961 – 3966. doi: 10.1016/j.tet.2009.03.046.

6. Hohne, A.; Yu, L.; Mu, L.; Reiher, M.; Voigtmann, U.; Klar, U.; Graham, K.; Schubiger, P. A.; Ametamey, S. M. *Chemistry Eur. J.* **2009**, *15*, 3736-3743. doi: 10.1002/chem.200802437.

7. Chantaud, C.; Xu, K.; Huang, H.; Wang Q. *J. Polym. Sci. Part A* **2010**, *48*, 4800-4810. doi: 10.1002/pola.24271.

8. Kavala, V.; Samal, A. K.; Patel, B. K. *Arkivoc* **2005**, *i*, 20-29. ISSN 1424-6376.

9. Magyar, Á.; Nagy, B.; Hell, Z. *Catal. Lett.* **2015**, *145*, 1876-1879. doi: 10.1007/s10562-015-1590-8.

10. Williams, D. B. G.; Simelane, S. B.; Lawton, M.; Kinfe, H. H. *Tetrahedron* **2010**, *66*, 4573-4576. doi: 10.1016/j.tet.2010.04.053.

11. Kumar, P.; Dinesh, C. U.; Reddy, R. S.; Pandey, B. *Synthesis* **1993**, 1069-1070. doi: 10.1055/s-1993-25998.

12. Stephens, J. R:; Butler, P. L.; Clow, C. H.; Oswald, M. C.; Smith, R. C.; Mohan R. S. *Eur. J. Org. Chem.* **2003**, 3827-3831. doi: 10.1002/ejoc.200300295.

13. Morizawa, Y.; Mori, I.; Hiyama, T.; Nozaki, H. *Synthesis* **1981**, 899-901. doi: 10.1055/s-1981-29640.

14. Traore, M.; Maynadier, M.; Souard, F.; Choisnard, L.; Vial, H.; Wong, Y.-S. *J. Org. Chem.* **2011**, *76*, 1409-1417. doi: 10.1021/jo102414s.

15. Beganskiene, A.; Nikishkin, N. I.; Luck, R. L.; Urnezius, E. *Heteroatom Chem.* **2006**, *17*, 656-663. doi: 10.1002/hc.20251.

16. Dušek, J.; Sklenář, V.; Jonas, J. *Collect. Czech. Chern. Commun.* **1981**, *46*, 2912-2923. doi: 10.1135/cccc19812912.

17. Iwasaki, T.; Agura, K.; Maegawa, Y.; Hayashi, Y.; Ohshima, T.; Mashima, K. *Chemistry Eur. J.* **2010**, *16*, 11567-11571. doi: 10.1002/chem.201000960.

18. Nolis, P.; Gil, S.; Espinosa, J.-F; Parella, T. *Magn. Reson. Chem.* **2009**, *47*, 121 – 132. doi: 10.1002/mrc.2363.

19. Lanfranchi, D. A.; Blanc, M.-C.; Vellutini, M.; Bradesi, P.; Casanova, J.; Tom, F. *Magn. Reson. Chem.* **2008**, *46*, 1188–1194. doi: 10.1002/mrc.2330.
